# High-coverage metabolomics uncovers microbiota-driven biochemical landscape of interorgan transport and gut-brain communication in mice

**DOI:** 10.1038/s41467-021-26209-8

**Published:** 2021-10-19

**Authors:** Yunjia Lai, Chih-Wei Liu, Yifei Yang, Yun-Chung Hsiao, Hongyu Ru, Kun Lu

**Affiliations:** Department of Environmental Sciences and Engineering, Gillings School of Global Public Health, CB# 7431, University of North Carolina, Chapel Hill, NC 27599 United States

**Keywords:** Metabolomics, Microbiology, Biochemical networks, Biomarkers

## Abstract

The mammalian gut harbors a complex and dynamic microbial ecosystem: the microbiota. While emerging studies support that microbiota regulates brain function with a few molecular cues suggested, the overall biochemical landscape of the “microbiota-gut-brain axis” remains largely unclear. Here we use high-coverage metabolomics to comparatively profile feces, blood sera, and cerebral cortical brain tissues of germ-free C57BL/6 mice and their age-matched conventionally raised counterparts. Results revealed for all three matrices metabolomic signatures owing to microbiota, yielding hundreds of identified metabolites including 533 altered for feces, 231 for sera, and 58 for brain with numerous significantly enriched pathways involving aromatic amino acids and neurotransmitters. Multicompartmental comparative analyses single out microbiota-derived metabolites potentially implicated in interorgan transport and the gut-brain axis, as exemplified by indoxyl sulfate and trimethylamine-*N*-oxide. Gender-specific characteristics of these landscapes are discussed. Our findings may be valuable for future research probing microbial influences on host metabolism and gut-brain communication.

## Abstract (web summary)

The gut microbiota harbours neuroactive potential with links to neurological disorders. Here, the authors apply global metabolomics with an integrated annotation strategy to comparatively profile fecal, blood serum and cerebral cortical brain tissues of eight-week-old germ-free mice vs. age-matched specific-pathogen-free mice, providing a snapshot of the metabolome status linked to the gut-brain axis.

## Introduction

The mammalian body and particularly the gastrointestinal (GI) tract is inhabited by hundreds of trillions of microbes, collectively termed the microbiota^1^. Complex, dynamic, and metabolically active by nature, these commensal microbes have been discovered to constantly interact with the host as a crucial mediator for physiological processes spanning energy harvest^2^, immune cell development^3^, and gut epithelial homeostasis^4^. Interestingly, recent studies support that microbiota also harbors novel neuroactive potential with links to neurological and/or psychiatric disorders, as further encapsulated as the “microbiota-gut-brain axis”^5^. For example, using maternal immune activation (MIA) mouse, a model of autism spectrum disorder (ASD), Hsiao and colleagues discovered^6^ that both ASD-mimicking GI barrier defects and behavioral abnormalities MIA offspring exhibited were restored through colonizing human commensal *Bacteroidetes fragilis*, supporting a gut-microbiota-brain connection for autism. In a recent work by Valles-Colomer and colleagues^7^, analyses of a large human cohort correlating fecal metagenomic features with indicators of quality of life and depression identified microbial strains, pathways, and metabolites pertaining to mental health and gut-brain interaction, providing the first population-scale evidence linking microbiota to mental health outcomes.

Despite the emerging data, whether and how microbiota controls brain function remains largely undefined. It has been postulated that at least two routes are involved^8^, namely (i) the vagus nerves (neuronal) that connect the central nervous system (CNS) and enteric nervous system (ENS, the “second brain”) and (ii) the circulatory system (humoral) encompassing blood and lymphatic circulation. Gut microbes, in close proximity to numerous local neurons and immune cells, may either act on the ENS in situ to signal the CNS remotely, or more likely, they synthesize or transform molecular cues that can translocate from gut lumen to systemic circulation, and possibly cross the blood-brain barrier (BBB) and affect CNS directly^9^. Despite such interest, surprisingly, molecular underpinnings for such microbiota-gut-brain axis are unclear. Although there have been sporadic studies targeting a few microbial molecules or chemical classes in this regard, as represented by α-synuclein^10^, 3,4-dihydroxyphenylacetate^7^, and bile acids^11^, many of these still have yet to be validated as a gut-brain mediator.

In this work, we use high-coverage comparative metabolomics analyses combining targeted and untargeted annotation strategies to address this (Supplementary Fig. 1)^12,13^. We profile fecal, blood sera, and cerebral cortical brain tissues of 8-week-old germ-free (GF) C57BL/6 mice and their age-matched conventionally raised (CONV-R) specific-pathogen-free counterparts using high-resolution mass spectrometry (HRMS). We assess group patterns using univariate and multivariate statistics, annotate chemical structures of all distinct ion features through an integrated cheminformatic approach, and leverage a suite of statistical and data visualization tools for systematic comparisons across sample compartments. Here, we report 701 unique metabolites of differentiated GF/CONV-R profiles; potential mechanistic links of resident microbiota to the humoral gut-brain axis are examined through enrichment analyses, random forest classification, and metabolomic network analysis. This work presents datasets from a unique multi-metabolome perspective for probing microbial influences on mammalian interorgan transport and gut-blood-brain interaction.

## Results

### High-coverage metabolomics of GF vs. CONV-R mice

**Fig. 1:**
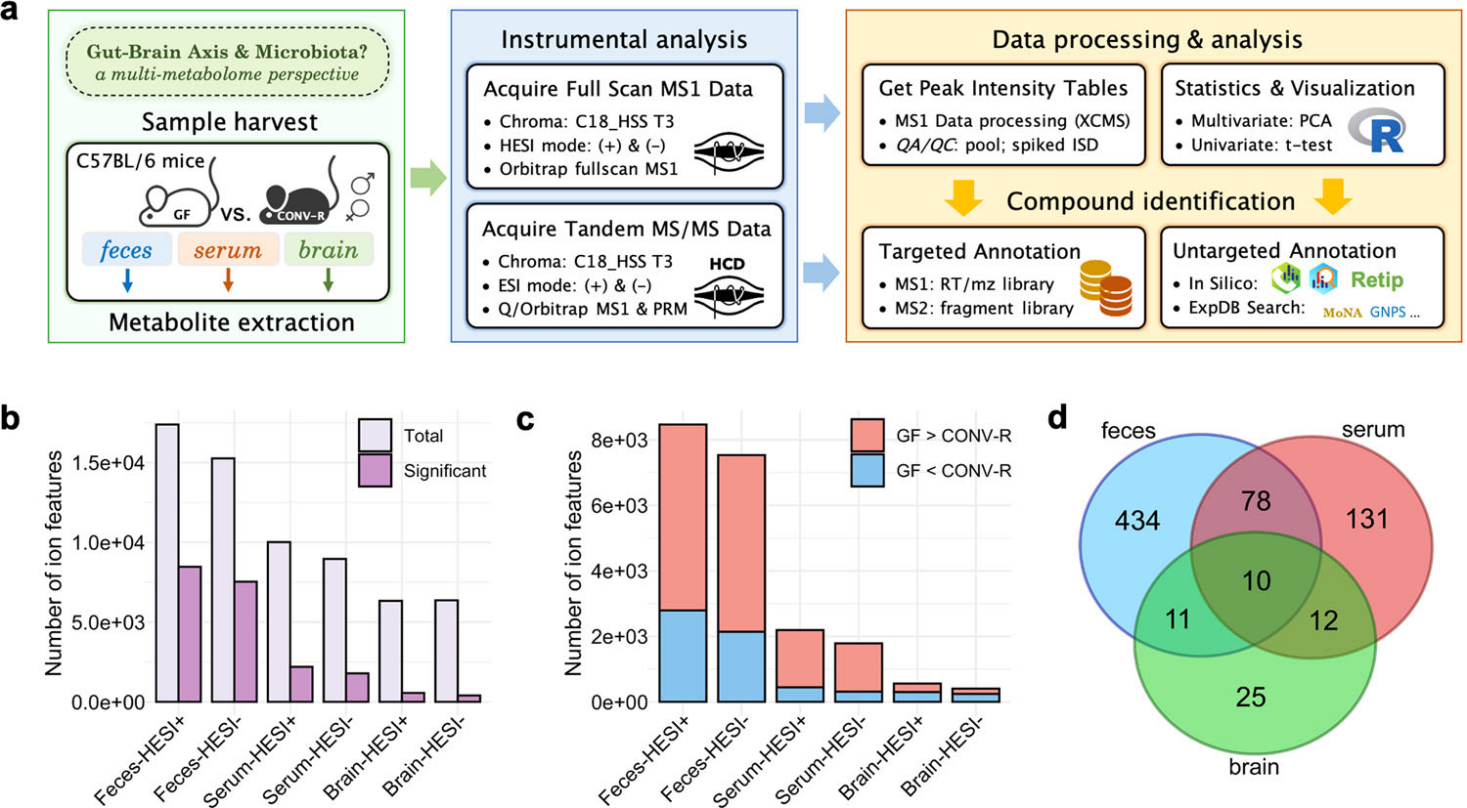
Overview of experimental approach and multi-metabolomics analyses. **a** Experimental workflow started with sample harvest and metabolite extraction of feces, blood sera, and cerebral cortical brain tissues from 8-week age-matched germ-free (GF, *N* = 12) and conventionally raised (CONV-R) specific-pathogen-free C57BL/6 mice (*N* = 12). A novel high-coverage metabolomics approach was used featuring orbitrap high-resolution mass spectrometry, targeted annotation based on an in-house mass spectral library, untargeted annotation using a streamlined cheminformatic pipeline for de novo structural dereplication, univariate and multivariate statistics, and data visualization. **b** Number of total and significant ion features (*p*-value < 0.01, fold change ≥ 1.5, two-sided Welch’s *t-*test) detected for feces, blood sera, and cortical brain tissues under HESI positive and negative modes of analysis. **c** Trend distribution of significantly altered ion features. **d** Venn diagram of all identified metabolites of GF/CONV-R difference among the three sample matrices. Chroma chromatography, HESI heated electrospray ionization, PRM parallel reaction monitoring, *QA*/*QC* quality assurance/quality control, ISD internal standard, PCA principal component analysis, RT/mz library retention time and mass-to-charge ratio pair library, ExpDB experimental database, MoNA MassBank of North America, GNPS The Global Natural Product Social Molecular Networking.

To uncover metabolites underlying microbiota-host interaction in light of gut-brain signaling, we conducted metabolomics, targeted and untargeted, on fecal matter, blood sera, and brain tissues (cerebral cortex slices) for 12 GF and 12 CONV-R C57BL/6 mice (8-week-old) (Fig. 1a). Using UHPLC-HESI-HRMS, we detected and aligned ion features into master peak tables for each sample type based on MS1 full-scan data, generating total ion feature numbers ranging from highest 17,386 for feces (heated ESI+) to lowest 6,334 for brain tissues (heated ESI+) (Fig. 1b). We statistically assessed these tables to screen for features of GF/CONV-R difference (fold change ≥ 1.5, *p* value < 0.01, Welch’s *t*-test) (Fig. 1b, c), yielding a total of 20,939 significant ion features including 16,001 for feces, 3,977 for sera, and 961 for brain tissues. Tandem mass spectra were acquired for all these features, and the highest metabolome coverage possible was achieved through a cheminformatic pipeline incorporating targeted and untargeted annotation procedures (Fig. 1a; Supplementary Fig. 1). The targeted procedure matched the unknown against an in-house spectral library containing retention time, accurate mass, and MS2 characteristic fragments for 423 authentic chemical standards of canonical pathway metabolites and 46 literature-reported microbial by-products including neurotransmitters (Supplementary Data 1). Meanwhile, untargeted analyses entailed validated rules and tools for formula generation^14^, structural dereplication^15^, and machine learning-based retention time prediction^16^. We manually curated and combined identification results from both procedures, yielding confidently annotated 533 fecal metabolites, 231 serum metabolites, and 58 metabolites of either level 1 confirmed structure or level 2 probable structure^17^ (Fig. 1d; Supplementary Data 2, 4).

### Microbiota as a master regulator of gastrointestinal metabolism

**Fig. 2:**
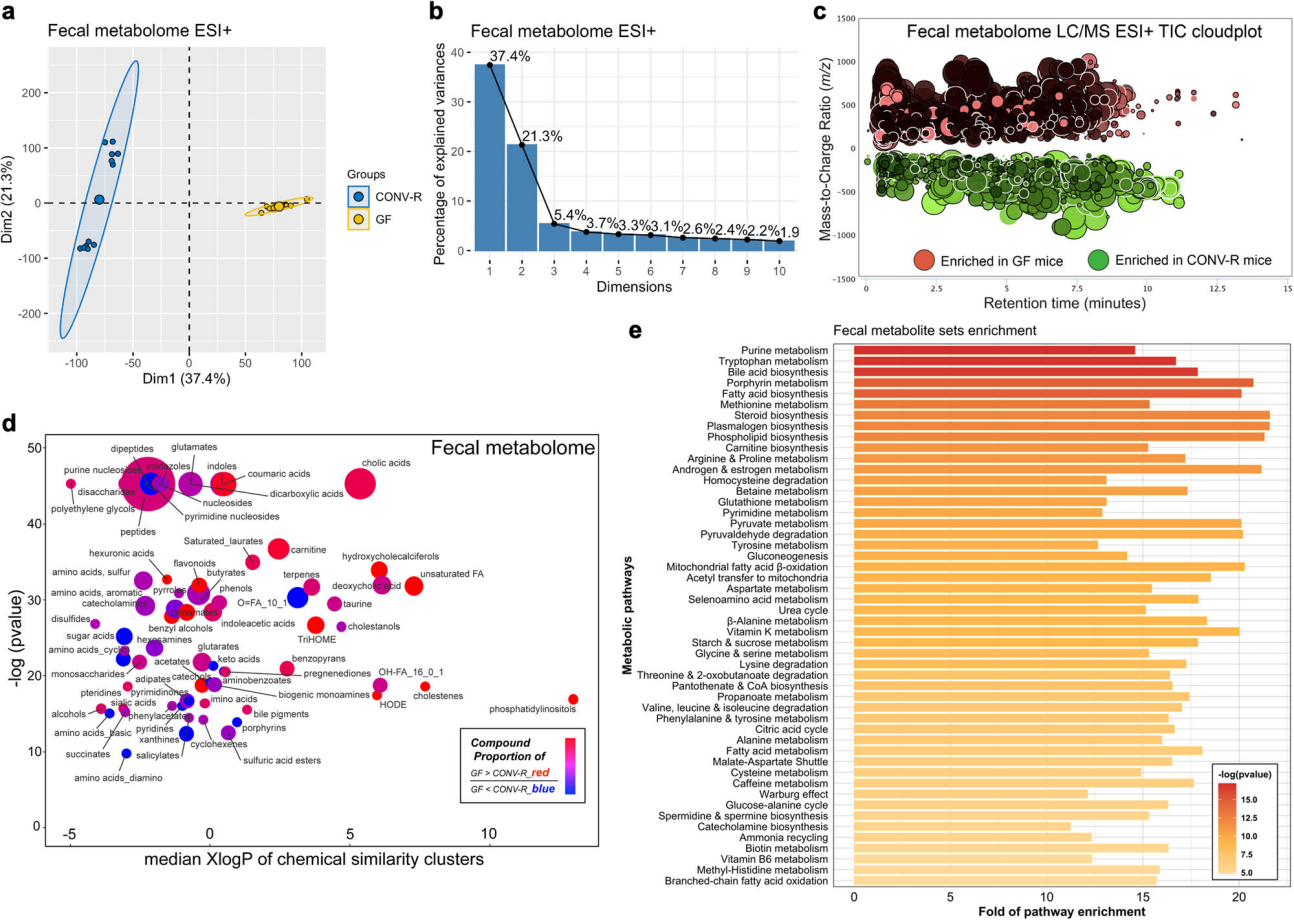
Distinct fecal metabolome profiles owing to the presence of microbiota. **a, b** Principal component analysis score plot (**a**) and scree plot (**b**) for assessing fecal metabolomic data comparing GF (*N* = 12) and CONV-R mice (*N* = 12) under heated ESI+ mode. **c** Metabolomic total ion chromatogram cloudplot of significant ion features in feces between groups (*p* value < 0.01, fold change ≥ 1.5, two-sided Welch’s *t* test) using heated ESI+ data as an example; larger circle size indicated larger fold change values ranging from 1.5 to 5300.6. **d** Chemical similarity enrichment analysis (ChemRICH) clustering of 533 identified altered fecal metabolites by chemical similarity with *x*-axis of mediation logarithmic additive octanol-water partition coefficients (XlogP) and *y*-axis for sets statistical significance based on the Kolmogorov–Smirnov test; the node size depicted total compound numbers for each cluster set and node color scale the proportion of GF-enriched vs. CONV-R enriched metabolites. **e** Quantitative metabolite set enrichment analysis (qMSEA) based on 99 a priori defined sets of metabolites identified a total of 71 significantly perturbed fecal metabolic pathways (adjusted *p* < 0.05) with top 50 shown. Dim dimension, LC/MS liquid chromatography-mass spectrometry, TIC total ion chromatogram, TriHOME trihydroxyoctadecenoic acid, FA fatty acid, HODE hydroxyoctadecadienoic acid, CoA coenzyme A.

Because the gut is the largest niche for commensal microbes and houses most critical sites of digestion, homeostasis, and immunity, gut lumen metabolomes represent a unique avenue for probing microbiota-host interaction^18^. Our metabolomics revealed distinct fecal patterns due to microbiota, as illustrated in principal component analysis (PCA) (Fig. 2a, b) and metabolomic total ion chromatogram cloudplot (Fig. 2c). Note that the 16,001 distinct fecal features accounted for 49.0% of the total detected. High-coverage cheminformatics successfully resolved 533 fecal metabolites from these features.

To gain a “landscape” view, chemical similarity enrichment analysis (ChemRICH)^19^ was performed. Seventy chemical classes were clustered, covering a wide lipophilicity range with varied compound numbers (node size) and overall trends of change (node color); for the color spectrum of node, red indicated that among the metabolites of GF/CONV-R difference, GF-enriched ones outnumbered those increased in CONV-R and/or overall embraced larger fold changes (Fig. 2d; Supplementary Data 5, 6). Most significantly enriched were cholic acids, oligopeptides, glutamates, indoles, and nucleosides, followed by other amino acids (e.g., sulfur, aromatic), lipids (e.g., acylcarnitines, fatty acids), and neurotransmitter families including taurine and catecholamines. To interpret in the context of biologic pathways, we conducted quantitative metabolite sets enrichment analysis (qMSEA) based on 99 a priori defined sets of metabolites in the Small Molecule Pathway Database (SMPDB)^20,21^. We identified 71 microbiota-perturbed pathways in the GI tract (adjusted *p* < 0.05) (Supplementary Data 7) with top 50 plotted (Fig. 2e). Our analyses enriched purines, tryptophan metabolism, bile acid biosynthesis, porphyrin metabolism, and fatty acid metabolism, alongside numerous other amino acids (e.g., arginine, proline, tyrosine, phenylalanine, aspartate, glycine, serine, lysine, and branched-chain amino acids), lipid metabolism (e.g., phospholipids, carnitines, and sex steroid hormones), cofactors (e.g., vitamin K, vitamin B_5_, biotin, vitamin B_3_, and vitamin B_6_), and polyamine metabolism (e.g., spermidine), etc. Both analyses confirmed for microbiota as a master regulator of gut metabolism, in protein metabolism (over 100 oligopeptides altered), energy harvest (e.g., fatty acid β-oxidation, carnitine shuttle, citric acid cycle, and gluconeogenesis), and numerous signaling pathways that involved amino acids and lipids as substrates or ligands.

**Fig. 3:**
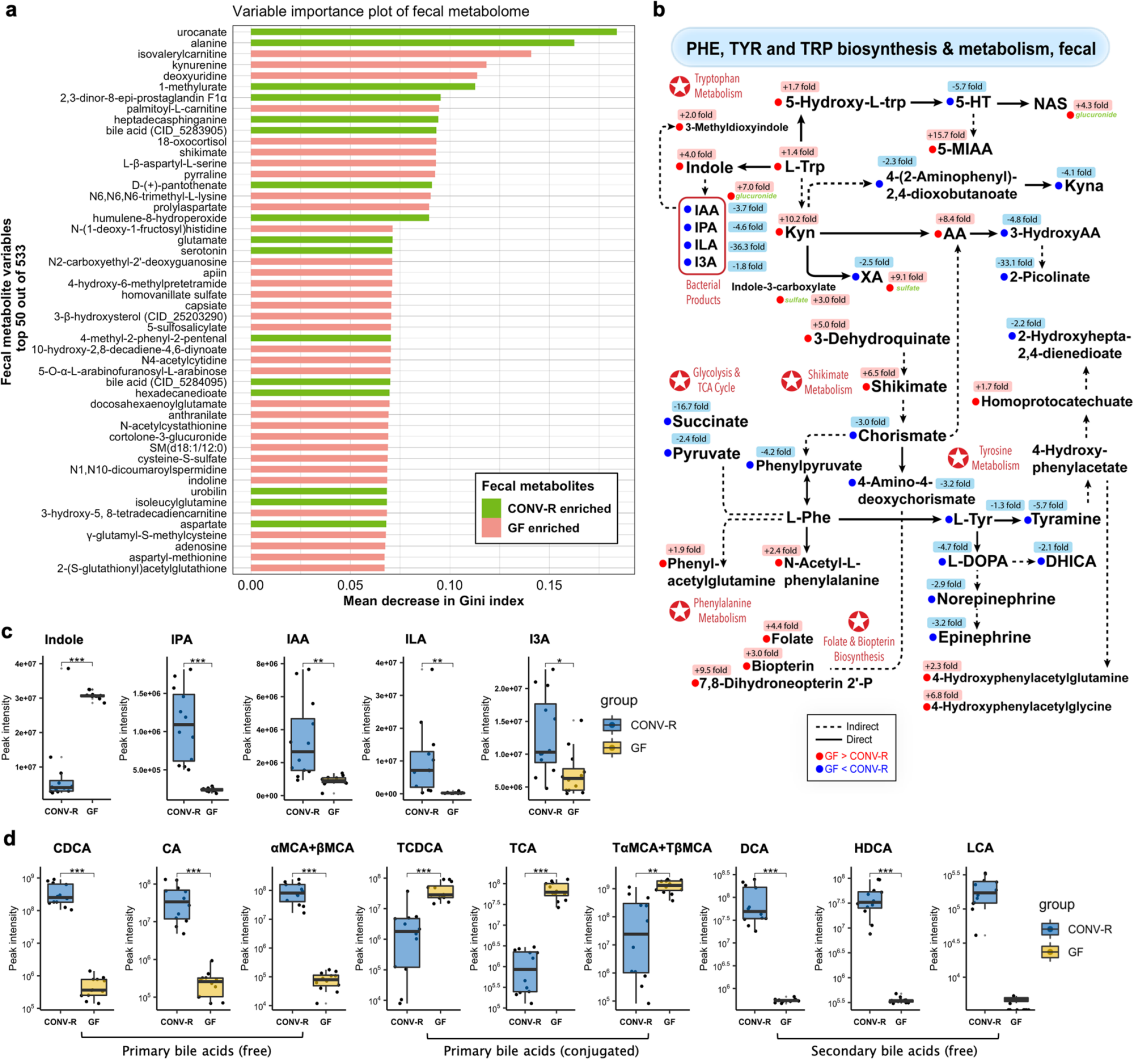
Select high-impact gut metabolites and pathways as regulated by microbiota. **a** Variable importance plot of top 50 fecal metabolites (*y*-axis) ranked by contribution to mean decrease accuracy of Gini coefficient (*x*-axis) in the random forest model for discerning group difference. **b** Diagram summary of perturbed fecal pathways of phenylalanine, tyrosine, and tryptophan biosynthesis and metabolism, with significantly altered metabolites labeled in red (GF>CONV-R) or blue (GF<CONV-R) (two-sided Welch’s *t*-test, fold change ≥ 1.5, *p* < 0.01). **c, d** Box and Whisker plots of fecal indoles (**c**) and bile acid profiles (**d**) as synthesized or mediated by microbiota, with the box ranging from the first quartile to the third while the whiskers going from each quartile to the minimum or maximum (GF, *N* = 12; CONV-R, *N* = 12), *p < 0.05, **p < 0.01, ***p < 0.001, ****p < 0.0001, two-sided Welch’s *t*-test; exact *p* values and adjusted *p* values (i.e., *q* values) are provided in Supplementary Data 2. SM sphingomyelin, Phe L-phenylalanine, Tyr L-tyrosine, Trp L-tryptophan, 5-HT 5-hydroxytryptamine, NAS N-acetylserotonin, 5-MIAA 5-methoxyindole-3-acetate, Kyna kynurenate, Kyn L-kynurenine, AA anthranilate, XA xanthurenate, IAA indole-3-acetate, IPA indole-3-propionate, ILA indole-3-lactate, I3A indole-3-carboxaldehyde, L-DOPA L-3,4-dihydroxyphenylalanine, DHICA 5,6-dihydroxyindole-2-carboxylate, CDCA chenodeoxycholate, CA cholate, αMCA α-muricholate, TCDCA taurochenodeoxycholate, TCA taurocholate, TαMCA tauro α-muricholate, DCA deoxycholate, HDCA hyodeoxycholate, LCA lithocholate.

We turned to individual fecal metabolites and select pathways for in-depth analyses. Using a random forest model, altered fecal metabolites were ranked by their relative contribution to group separation with top 50 shown in a variable importance plot (VIP) (Fig. 3a). Top-ranked members were structurally diverse, indicating a multifaceted and complex nature of the microbiome-metabolome network. We specifically focused on aromatic amino acid pathways because they have confirmed microbial involvement and neuromodulatory activities^22–24^, enriched with tryptophan metabolism being the second most significant (Fig. 2e), represented major compound classes perturbed (in addition to those for energy harvest and protein metabolism) (Fig. 2d), and included multiple metabolite members highly ranked in the random forest classification model, including kynurenine (4th), shikimate (12th), and serotonin (21st) (Fig. 3a). In the integrated view (Fig. 3b), all three tryptophan catabolic fluxes were enhanced when microbiota was present, as characterized by (i) reduced tryptophan pools (1.4-fold), (ii) decrease levels of kynurenine, 5-hydroxy-L-tryptophan, and indole, and (iii) increased levels of serotonin (5-HT) (5.7-fold), kynurenate (4.1-fold), and indole derivatives of 1.8–36.3-fold changes including indole-3-propionate (IPA), indole-3-acetate (IAA), indole-3-lactate (ILA) and indole-3-carboxaldehyde (I3A) (Fig. 3b, c). We also observed distinct bile acid patterns with fold changes as large as three orders of magnitude (Fig. 3d; Supplementary Data 2). Note that free-form primary bile acids such as chenodeoxycholate, cholate, muricholates (αMCA+βMCA) were markedly higher in CONV-R feces, while their taurine- or glycine- conjugates had an opposite trend. Correspondingly, secondary bile acids were found to be much enriched in CONV-R feces than in GF’s (Fig. 3d; Supplementary Data 2).

### Microbiota extensively mediates gut neurotransmitter production and transformation

**Fig. 4:**
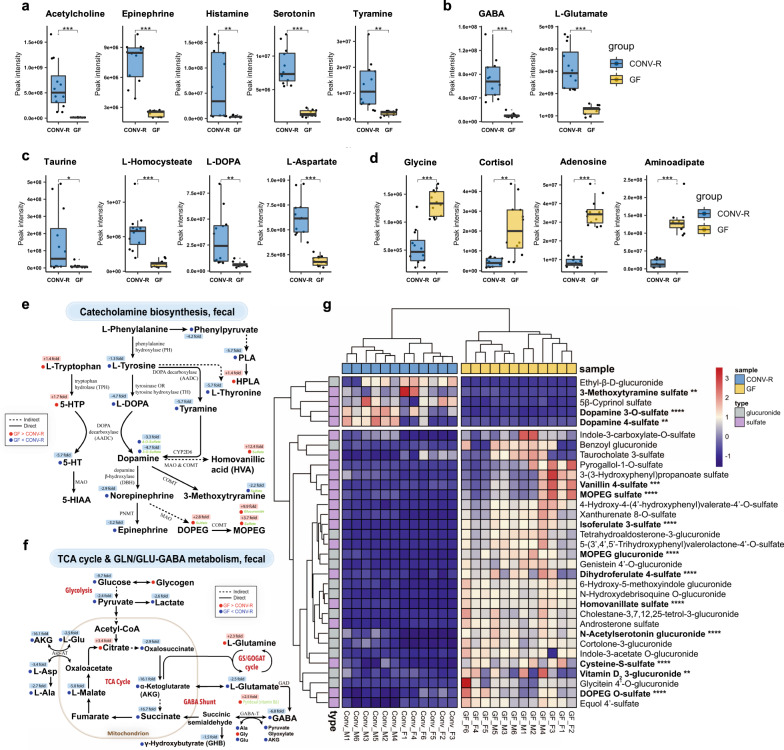
Microbiota extensively mediates neurotransmitter production, deconjugation and transformation in the GI tract. **a–d** Box and Whisker plots of fecal Class A (Rhodopsin-like) GPCR amine neurotransmitters (**a**), fecal Class C metabotropic neurotransmitter L-glutamate and GABA (**b**), CONV-R enriched neurotransmitters and related molecules (**c**) and GF-enriched neurotransmitters and related molecules (**d**), with the box ranging from the first quartile to the third while the whiskers going from each quartile to the minimum or maximum (GF, *N* = 12; CONV-R, *N* = 12), *p < 0.05, **p < 0.01, ***p < 0.001, ****p < 0.0001, two-sided Welch’s *t* test; exact *p*-values and adjusted *p* values (i.e., *q* values) are provided in Supplementary Data 2. (**e**) Diagram summary of altered fecal catecholamine biosynthetic pathways due to microbiota, with the significantly altered metabolites labeled in red (GF>CONV-R) or blue (GF<CONV-R) (two-sided Welch’s *t*-test, fold change ≥ 1.5, *p* < 0.01). **f** Diagram summary of altered fecal (glutamine-)glutamate-GABA metabolism coupling with citric acid cycle due to microbiota, with the significantly altered metabolites labeled in red (GF>CONV-R) or blue (GF<CONV-R) (Welch’s *t*-test, fold change ≥ 1.5, *p* < 0.01). **g** Heatmap clustering of distinct fecal metabolites from Phase II reaction; glucuronides or sulfates of neurotransmitters or related compounds were labeled by bold texts with asterisks showing statistical significance. GABA γ-aminobutyrate, 5-HTP 5-hydroxy-L-tryptophan, 5-HT 5-hydroxytryptamine, 5-HIAA 5-hydroxyindoleacetate, PLA DL-3-phenyllactate, HPLA DL-p-hydroxyphenyllactate, DOPEG 3,4-dihydroxyphenylglycol, MOPEG 3-methoxy-4-hydroxyphenylglycol, MAO monoamine oxidase, CYP2D6 cytochrome P450 2D6, COMT catechol-O-methyltransferase, TCA cycle tricarboxylic acid cycle, Gln/Glu-GABA metabolism glutamine/glutamate-γ-aminobutyrate metabolism, AKG α-ketoglutarate, L-Asp L-asparagine, L-Ala, L-alanine, GS/GOGAT cycle glutamine synthase/glutamate:2-oxoglutarate aminotransferase cycle, Gly L-glycine, GABA-T 4-aminobutyrate transaminase, GAD glutamic acid decarboxylase.

The discovery that our commensal microbiota comprises innumerable neurotransmitter producers has recently led to exciting hypotheses questioning their effects on host neurotransmission, gut-brain signaling, and mental health outcomes^25,26^. Here, we offer a complete analysis of fecal neurotransmitter profiles comparing GF and CONV-R mice. As selectively shown (Fig. 4a–d), we found for a number of neurotransmitters much-elevated levels in the presence of microbiota, spanning Class A (Rhodopsin-like) G-protein coupled receptor pathway (GPCR) amines including acetylcholine, epinephrine, histamine, serotonin, and tyramine (Fig. 4a), Class C metabotropic neurotransmitters L-glutamate and GABA (Fig. 4b) to neurotransmitters (or their precursors) of other channels or receptors, e.g., taurine, L-homocysteate, L-3,4-dihydrophenylalanine (L-DOPA) (Fig. 4c). Many neurotransmitters were virtually absent in GF feces, as indicated by peak intensities as low as detection limits even on ultra-sensitive mass spectrometry assays of ours. This suggests an essential role of resident microbiota for neurotransmitter production. Several neuroendocrine signaling molecules, namely glycine, cortisol, adenosine, and 2-aminoadipate embraced an opposite trend (Fig. 4d), suggesting endogenous sources other than influences from microbiota.

We took turns to specifically examine two major neurotransmitter pathways, namely catecholamine biosynthesis (Fig. 4e) and the glutamine/glutamate-GABA cycle (Fig. 4f), since both have been suggested recently as potential routes for microbes to modulate host neurotransmission extending beyond the GI tract to the entire body. Catecholamines, namely dopamine, norepinephrine, and epinephrine, are biogenic amine neurotransmitters with crucial functions in motivation, reward, and hedonistic regulation^27^. Consistent with previous data^28^, our results showed markedly higher fecal levels of norepinephrine (2.9-fold), epinephrine (3.2-fold), as well as two dopamine precursors tyramine (5.7-fold) and L-DOPA (4.7-fold). Though fecal dopamine levels manifested no statistical difference, we found two related sulfate-conjugates (dopamine-3-O-sulfate and dopamine-4-sulfate) substantially elevated when microbiota was present (Fig. 4e), warranting future analyses. Glutamine/glutamate-GABA metabolism, coupling with much altered tricarboxylic acid (TCA) cycle, was also perturbed in the gut, as represented by decreased L-glutamine (2.3-fold) and elevated L-glutamate (2.5-fold) and GABA (6.8-fold) with the presence of microbiota (Fig. 4f).

Unexpectedly, we observed a realm of conjugated compounds altered owing to microbiota, involving glucuronides and sulfates of neurotransmitters (Fig. 4g). We constructed a heatmap of them, with 28 enriched in CONV-R feces and five with opposite trends (Fig. 4g). Based on extensive literature search, we highlighted those with neuromodulatory properties in bold texts, including 11 compounds much diminished (e.g., N-acetylserotonin glucuronide) and three enriched (e.g., dopamine 4-sulfate) when microbiota was present, alongside other conjugates of diet-derived flavonoids (e.g., genistein 4’-O-glucuronide), bile acids (e.g., taurocholate 3-sulfate), and indole derivatives (e.g., indole-3-carboxylate-O-sulfate, indole-3-acetate O-glucuronide) (Fig. 4g). These data together support that the present microbiota plays an integral role in neurotransmitter metabolism in the local gut lumen in terms of production, transformation, and bioavailability, warranting future efforts to delineate microbial species-level contributions in a time- and space-specific manner.

### Microbial impacts on circulating blood metabolism and fecal-blood exchange

**Fig. 5:**
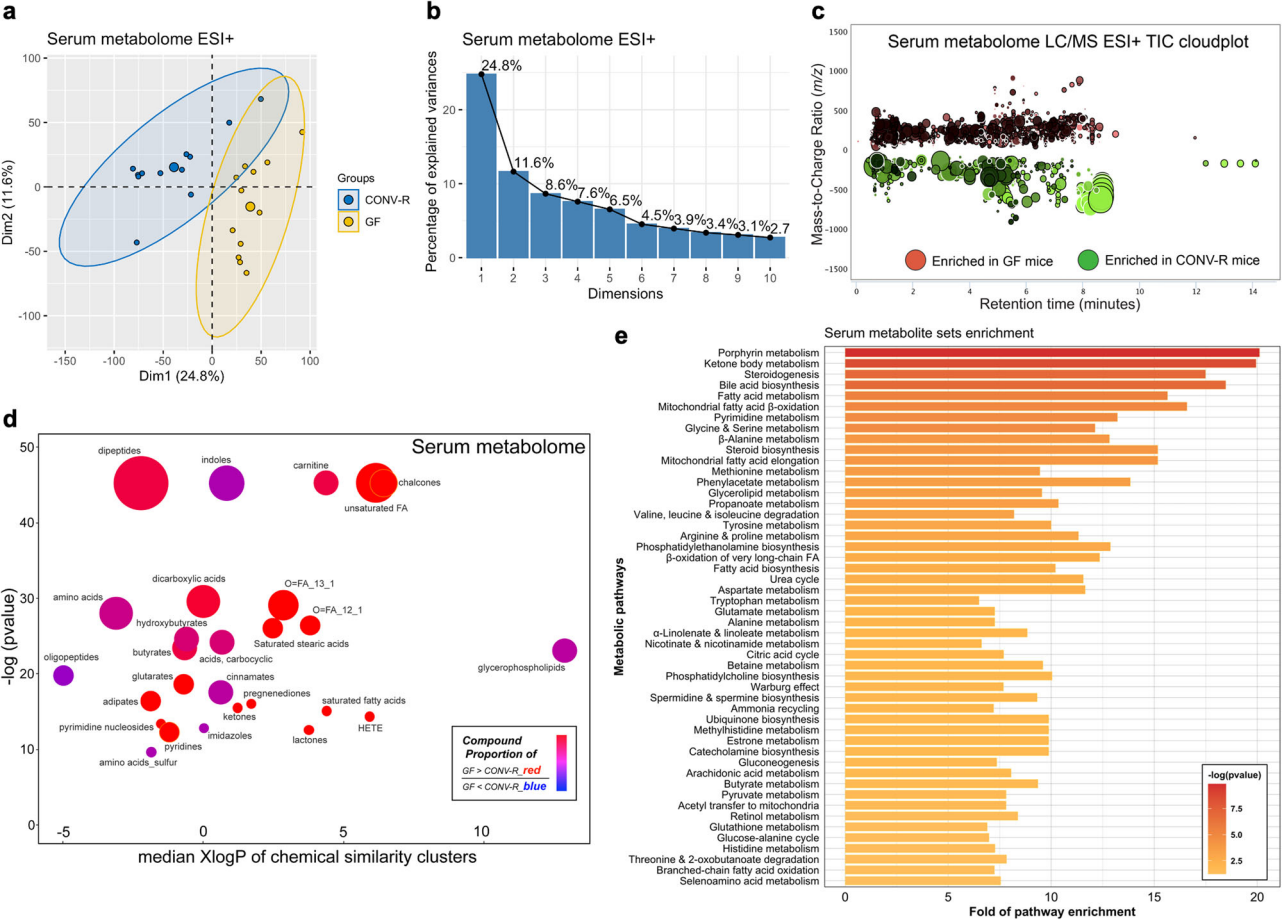
Global effects of microbiota on host circulating blood metabolism. **a, b** Principal component analysis score plot (**a**) and scree loading plot (**b**) for assessing serum metabolomic data comparing GF (*N* = 12) and CONV-R mice (*N* = 12) under heated ESI+ mode. **c** Metabolomic total ion chromatogram cloudplot of significant features in serum between groups (*p* value < 0.01, fold change ≥ 1.5, two-sided Welch’s *t* test) using heated ESI+ as an example; larger circle size indicated larger fold change values ranging from 1.5-1,347.1. **d** Chemical similarity enrichment analysis (ChemRICH) clustering of 231 identified altered serum metabolites by chemical similarity with *x*-axis of mediation logarithmic additive octanol-water partition coefficients (XlogP) and *y*-axis for sets statistical significance based on the Kolmogorov–Smirnov test; the node size depicted total compound numbers for each cluster set and node color scale the proportion of GF-enriched vs. CONV-R enriched metabolites. **e** Quantitative metabolite set enrichment analysis (qMSEA) based on 99 a priori defined sets of metabolites identified a total of 57 significantly perturbed serum metabolic pathways (adjusted *p* < 0.05) with top 50 shown. LC/MS liquid chromatography-mass spectrometry, TIC total ion chromatogram, HETE 5-hydroxyeicosatetraenoic acid, FA fatty acid.

With detailing of microbiota’s control over gut metabolism and neurotransmitter profiles, the question arises as to whether these local effects will propagate from gut to peripheral organs. To investigate this, we profiled the circulating blood sera of GF and CONV-R mice. We observed systemic perturbations, though not as striking as the fecal data, for serum metabolome due to microbiota (Fig. 5a–c), with 3,977 significant ion features detected that accounted for 21.0% of total aligned features. We successfully resolved 231 unique structures from these altered features (Supplementary Data 3).

To infer, we performed chemical similarity enrichment and quantitative pathway enrichment analyses. ChemRICH plot clustered the serum metabolites into 27 chemical classes, spanning carnitines, dipeptides, indoles alongside lipid species, amino acids, and organic acids (Fig. 5d; Supplementary Data 8, 9). We discovered that 175 out of the total 231 metabolites (75.8%) were downregulated in the presence of microbiota, also indicated by the prevailing purple-to-red colors (Fig. 5d). qMSEA analysis revealed for circulating blood 61 significantly enriched pathways (adjusted *p* < 0.05) (Supplementary Data 10), with top 50 shown (Fig. 5e). In line with fecal data, top enriched serum pathways spanned from porphyrin metabolism, ketone body metabolism, bile acid biosynthesis to numerous amino acid metabolism, including the aromatic amino acid family (Fig. 5e). The results show that microbiota has systemic effects and can modulate peripheral blood circulation, raising possibilities of a microbiota-gut-brain axis through humoral transport of molecular cues in vivo.

**Fig. 6:**
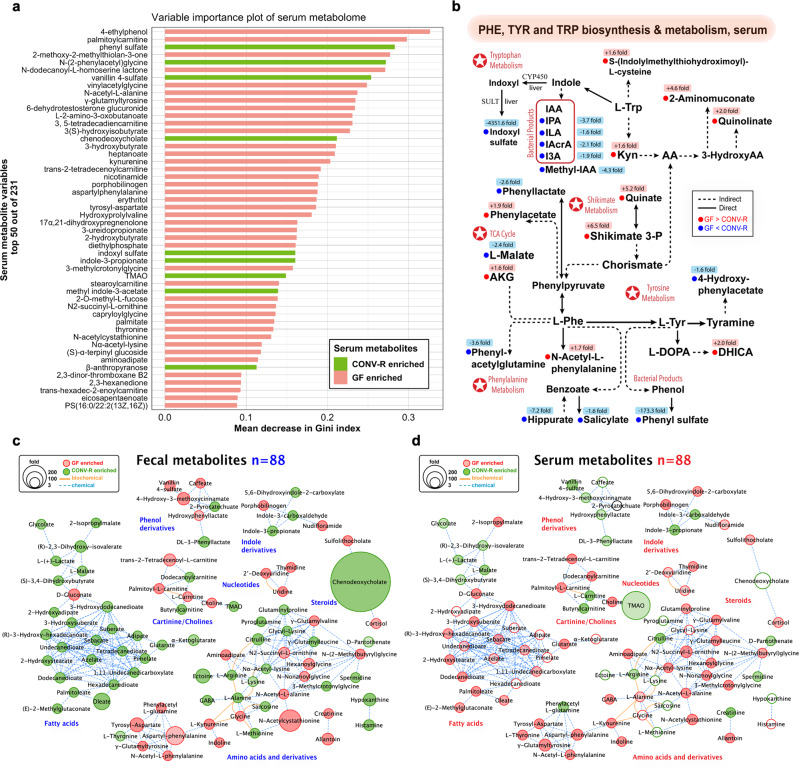
Select high-impact metabolites and pathways in circulating blood and their enterohepatic transport. **a** Variable importance plot of top 50 serum metabolites (*y*-axis) ranked by contribution to mean decrease accuracy of Gini coefficient (*x*-axis) in the random forest model for discerning group difference. **b** Diagram summary of perturbed serum pathways of phenylalanine, tyrosine and tryptophan biosynthesis and metabolism. **c, d** MetaMapp metabolomic networks of 88 metabolite pairs altered in feces (**c**) and serum (**d**), with nodes representing individual metabolites, edges for biochemical (KEGG reactant pairs) and chemical (Tanimoto coefficient > 0.7) relationships, and lower transparency for lower adjusted *p* values (<0.05, two-sided Welch’s *t*-test). TMAO trimethylamine N-oxide, PS phosphatidylserine, Phe L-phenylalanine, Tyr L-tyrosine, Trp L-tryptophan, Kyn L-kynurenine, AA anthranilate, IAA indole-3-acetate, IPA indole-3-propionate, ILA indole-3-lactate, IArcA indole-3-acrylate, I3A indole-3-carboxaldehyde, Shikimate 3-P shikimate 3-phosphate, DHICA 5,6-dihydroxyindole-2-carboxylate, AKG α-ketoglutarate, L-DOPA L-3,4-dihydroxyphenylalanine, SULT sulfotransferase, CYP450 cytochrome 450, TCA cycle tricarboxylic acid cycle.

Similar to fecal data, we focused on individual serum metabolites for in-depth analyses to delineate potential gut-blood network and neuroendocrine pathways. Random forest analyses ranked all altered 231 serum metabolites by contribution to group separation, with top 50 shown (Fig. 6a). In addition to metabolites for energy processes (e.g., acylcarnitines, oligopeptides, ketone bodies), we noted a realm of microbial products in sera. For example, the top-ranked 4-ethylphenol, is a known tyrosine metabolite synthesized by bacteria^29,30^. Phenyl sulfate ranked third with surging levels in CONV-R sera (173.33-fold), is a confirmed gut-microbiota-derived uremic toxin contributing to albuminuria in diabetic kidney disease^31^ (Fig. 6a; Supplementary Data 3). Another phenolic derivative N-(2-phenylacetyl)glycine (PAG), with 10.5-fold elevation with microbiota, was ranked the fifth place. In the VIP chart, we also observed several tryptophan indole derivatives enriched in CONV-R serum. These included indoxyl sulfate (4,351.6-fold), IPA (7.3-fold), methyl indole-3-acetate (4.3-fold) alongside other indoles (Supplementary Fig. 2a). The results together support that microbiota’s systemic effects on the host involve humoral transport of microbial molecular cues.

To detail the microbiota-serum metabolome network, we focused on aromatic amino acid pathways (Fig. 6b) with multiple top-ranked phenolics and indoles on the VIP chart (Fig. 6a). Compared with fecal data (Fig. 3b), we observed for CONV-R sera overall a consistent pattern, such as enhanced fluxes of tryptophan catabolism featuring decreased kynurenine and enriched indoles. That said, interestingly, we noted that serum serotonin levels, though at high peak intensity (area, 10^8^), were not statistically different between the CONV-R and GF mice, which was in conflict with studies illuminating microbiota’s regulation of peripheral serotonin levels^32^. We re-evaluated our data and discovered that serum serotonin levels were markedly higher in male CONV-R mice than in male GF mice (*p* = 0.0022, pairwise Wilcoxon rank-sum test, with a CONV-R/GF fold change of 1.6), while female mice exhibited no statistical difference (*p* = 0.18) (Supplementary Fig. 2b), suggesting gender as a potential factor in gut microbial effects on mammalian circulating serotonin levels. We also examined serum neurotransmitter and neuromodulatory compounds (Supplementary Fig. 2c) and noted that glycine, cortisol, and 2-aminoadipate shared a similar trend with fecal data (decreased in CONV-R mice). Whereas histamine and GABA embraced an opposite trend with markedly lower levels in CONV-R than in GF sera. Surprisingly, CONV-R-gut-enriched neurotransmitters such as acetylcholine, epinephrine, and taurine were either below detection levels in serum or exhibited no statistical difference between groups. The results together show that microbiota’s modulation of peripheral circulating neurotransmitters is strictly compartmentalized.

To further delineate gut-blood exchange, we focused on metabolites of GF/CONV-R difference shared by fecal and serum matrices. In total, 88 metabolite pairs are shown in MetaMapp network graphs^33^ for a side-by-side comparison (Fig. 6c, d). The network analyses generated nine compound clusters, including fatty acids, amino acids, and carnitines/choline. The overall change in serum was smaller than in feces, as indicated by generally smaller node sizes and larger adjusted *p*-values of Welch’s *t*-test (Fig. 6c, d). We noted that fatty acid pairs, including microbiota-derived adipate^34^ and sebacate^35^ embraced a contrasting gut-blood pattern, with 20 elevated in feces but lowered in serum for microbiota-harboring mice. Conversely, phenolics such as hydroxyphenyllactate and 4-hydroxy-3-methoxycinnamate were lower in feces but higher in blood sera for CONV-R mice. Apart from these two, other compound classes showed similar fecal-blood patterns of change, spanning amino acid derivatives (including indoles), nucleotides, steroids, and carnitines/choline. The results support that microbiota’s impacts on local gut epithelial homeostasis, energy harvest, and signaling can translocate and propagate to peripheral blood circulation, raising further possibilities of humoral transport and effects on host CNS.

### Integrated analyses of cerebral cortical brain, feces, and blood serum metabolomes

**Fig. 7:**
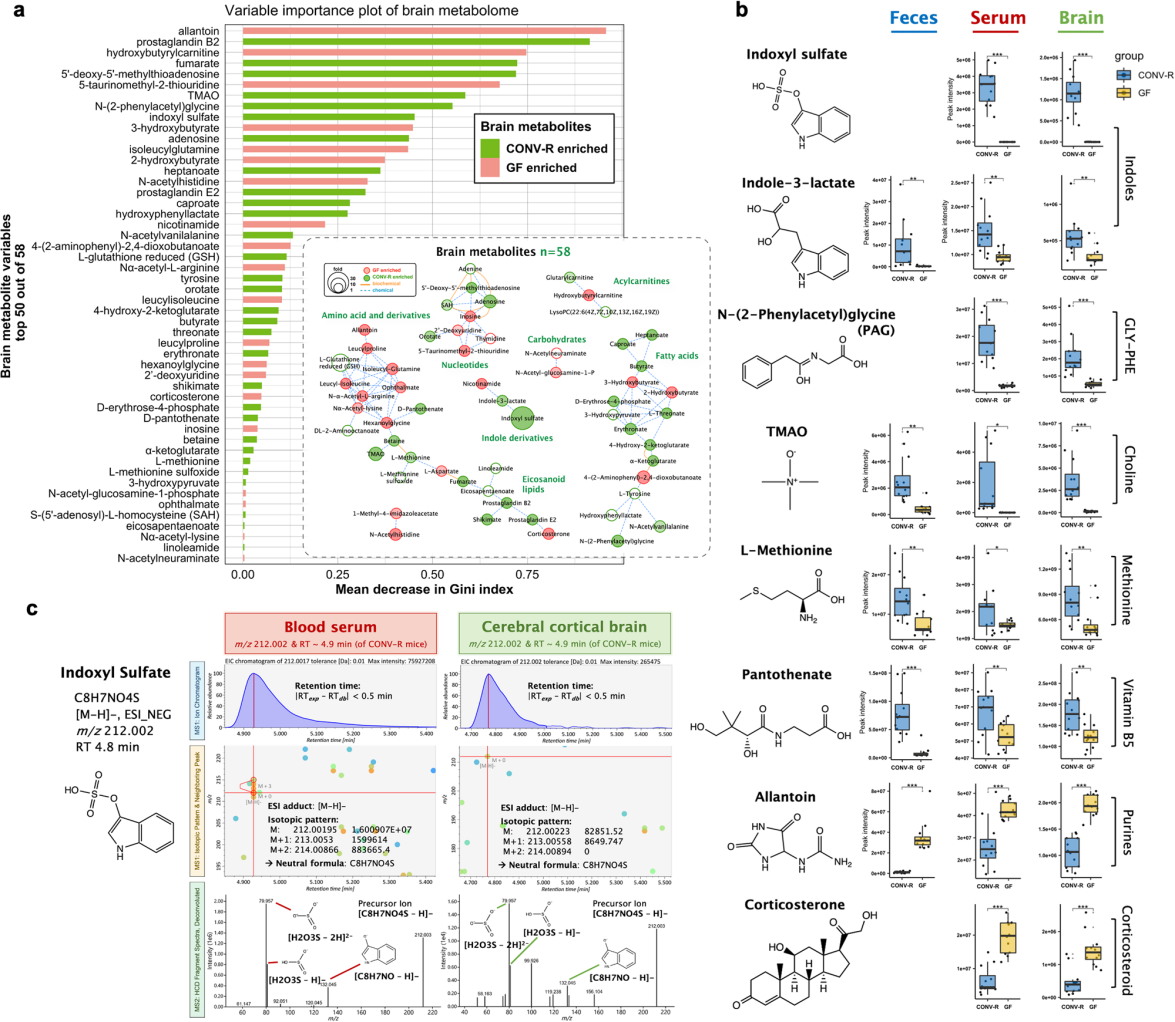
Metabolites of microbial neuroactive potential: integrated analyses of cerebral cortical brain, fecal and blood serum metabolomes. **a** Variable importance plot of top 50 brain metabolites (*y*-axis) ranked by contribution to mean decrease accuracy of Gini coefficient (*x*-axis) in the random forest model for discerning group difference; embedded was a MetaMapp network view of all 58 metabolites altered in cerebral cortical brain tissues owing to microbiota, with nodes representing individual metabolites, edges for biochemical (Kyoto Encyclopedia of Genes and Genomes, i.e., KEGG reactant pairs) and chemical (Tanimoto coefficient > 0.7) relationships and lower transparency for lower *p* values (<0.05, two-sided Welch’s *t* test). **b** Box and Whisker plots of select metabolites exhibiting systemic alterations across feces, blood sera, and cerebral cortical brain tissues as mediated by microbiota, with the box ranging from the first quartile to the third while the whiskers going from each quartile to the minimum or maximum (*n* = 24), *p < 0.05, **p < 0.01, *** < 0.001, ****p < 0.0001, two-sided Welch’s *t* test; exact *p* values and adjusted *p* values (i.e., *q* values) are provided in Supplementary Data 4. (**c**) Structural annotation of ion feature *m/z* 212.002 at the retention time of 4.9 min as indoxyl sulfate that was highly enriched in CONV-R blood sera (4351.6-fold) and cerebral cortical brain tissues (26.8-fold) compared with GF mice. PAG N-(2-phenylacetyl)glycine, TMAO trimethylamine N-oxide, Gly-Phe glycine-phenylalanine dipeptide, HCD higher-energy C-trap dissociation, EIC extracted ion chromatogram, *m/z* mass-to-charge ratios, RT_exp_ experimental retention time (from data), RT_db_ reference retention time (from chemical standard).

To further determine whether commensal microbiota modulates brain biochemistry, we profiled the metabolome of cerebral cortical brain tissues comparing GF and CONV-R mice. We focused on the cerebral cortex region because it is the largest site of neural integration (occupying over two-thirds of mammalian brain mass), carries pivotal roles in brain functions such as thinking, memory, language, and consciousness^36,37^, and importantly, cerebral cortical injuries are often linked to onset of mental disorders such as depression^38^ among which many also are common comorbidities to gut dysbiosis. In total, we identified 58 altered metabolites in cerebral cortical brain tissues (Supplementary Data 4). To interpret these, we constructed a network graph of all metabolites and ranked them based on a random forest model (Fig. 7a). The network showed changes in diverse compounds, as represented by indoles, amino acids, nucleotides, and fatty acids. On the VIP chart, we discovered an array of markers and pathways related to oxidative stress and reactive oxygen species (ROS) reaction. For example, the methionine/glutathione transsulfuration pathway was extensively perturbed in brain, as characterized by elevated levels of L-methionine (1.47-fold), S-(5’-adenosyl)-L-homocysteine (SAH, 1.30-fold), L-glutathione (GSH, 15.1-fold), and 5’-deoxy-5’-methylthioadenosine (1.62-fold) when microbiota was present (Supplementary Fig. 3)^39,40^. We also noted for CONV-R brain elevated levels of two eicosanoids prostaglandin B2 (1.8-fold) and E2 (1.7-fold), α-ketoglutarate (AKG, 1.4-fold) (a sensitive oxidative stress indicator) as well as the antioxidative ketone body 3-hydroxybutyrate (2.0-fold). In addition to generic markers, we discovered a range of microbiota-derived metabolites implicated in redox homeostasis. These spanned short-chain fatty acids (e.g., butyrate, caproate), indoles (e.g., indoxyl sulfate, indole-3-lactate), trimethylamine-*N*-oxide (TMAO), shikimate and phenylalanine derivatives (e.g., hydroxyphenyllactate, PAG), all elevated in CONV-R brain tissues compared with GF mice (Fig. 7a). The results support that commensal microbiota can largely mediate, directly or indirectly, redox homeostasis, energy metabolism, as well as neuronal signaling inside the mammalian CNS.

To further interpret in light of the gut-brain axis, we compared multiple matrices for an integrated analysis, with examples shown in boxplots (Fig. 7b). We noted under heated ESI- analysis mode, ion feature *m/z* 212.002 at the retention time of 4.9 minutes was much elevated in CONV-R mice while virtually absent in GF mice, with highest fold changes in both sera (4,351.6-fold) and brain tissues (26.8-fold) (Fig. 7b). In silico formula analyses determined the elemental composition of this ion feature to be C_8_H_6_NO_4_S ([M-H]-), based on which the neutral structure was dereplicated and confirmed as indoxyl sulfate (Fig. 7c). Indoxyl sulfate was a known uremic toxin and cardiotoxin that originated from host-microbiota metabolism of dietary tryptophan. Our results indicated that microbiota-derived indoxyl sulfate may cross the BBB and reach the CNS, which was consistent with two emerging studies^41,42^. Similar to indoxyl sulfate which has microbial relevance and shared trends across compartmental matrices, we also noted two other aromatic amino acid derivatives indole-3-lactate and PAG that were enriched in CONV-R mice. Other potential humoral pathways indicated in this study were exemplified for CONV-R mice by elevated levels of TMAO, methionine, vitamin B_5_ in all three matrices and decreased levels of allantoin and corticosterone in serum and brain tissues (Fig. 7b; Supplementary Data 1–4).

### Microbial rewiring of host biochemistry has gender-specific characteristics

**Fig. 8:**
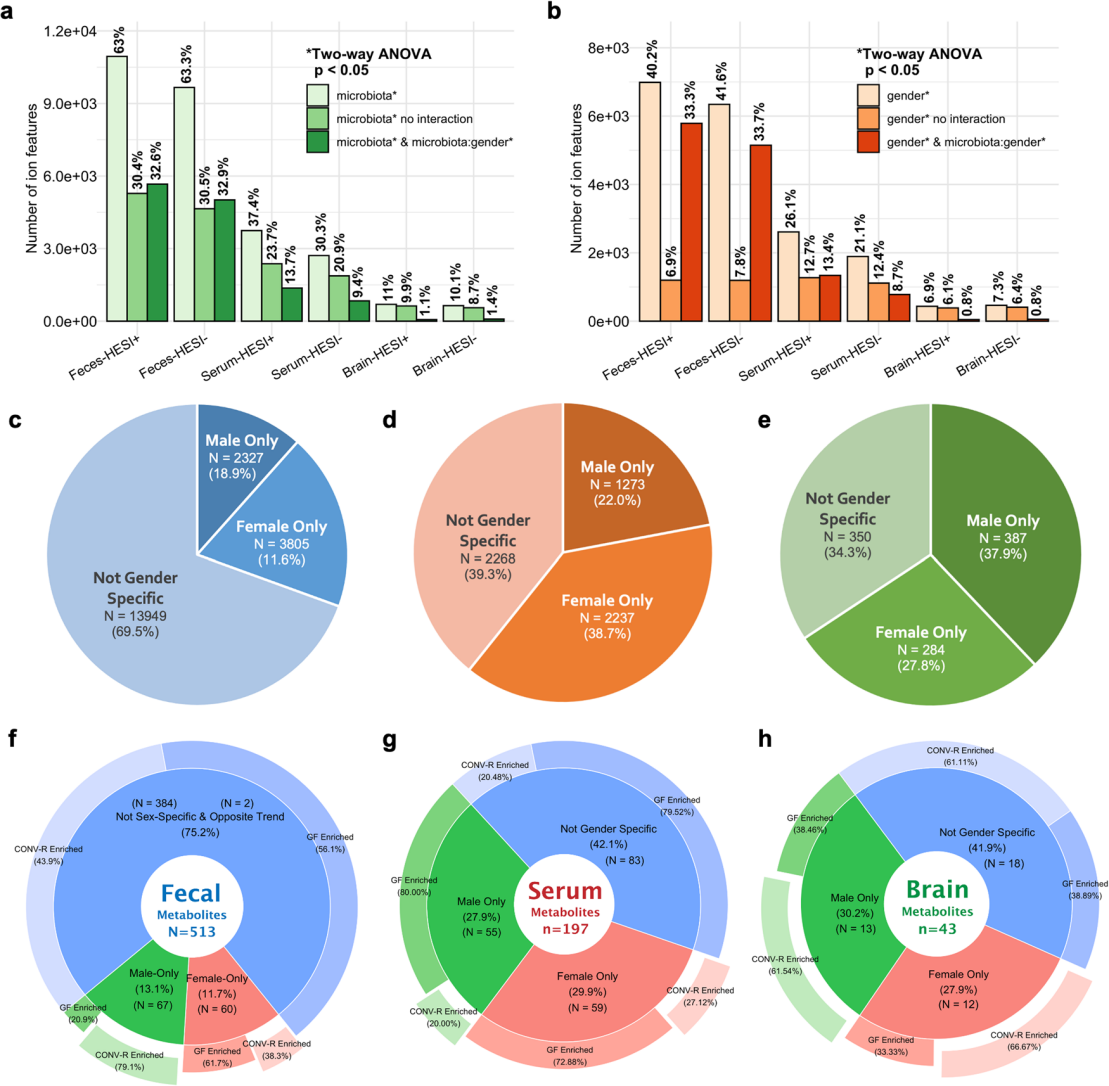
Microbial rewiring of host metabolism and gut-brain axis exhibits gender-specific characteristics. **a** Proportions of ion features with significant main effects of microbiota as determined by two-way analysis of variance (ANOVA) (adjusted *p* < 0.05). **b** Proportions of ion features with significant main effects of gender as determined by two-way ANOVA (adjusted *p* < 0.05). **c–e** Pie charts for gender-specific distribution of ion features in feces (**c**), sera (**d**), and cerebral cortical brain (**e**) combining both HESI positive and negative modes that had main effects of microbiota (two-way ANOVA, adjusted *p* < 0.05) while exhibiting statistical GF/CONV-R difference in at least one gender (post hoc Tukey’s HSD test, adjusted *p* < 0.05). **f–h** PieDonut charts for gender-specific distribution of the identified compounds in feces (**f**), sera (**g**), and cerebral cortical brain (h) as determined by two-way ANOVA (adjusted *p* < 0.05) and post hoc Tukey’s HSD test (adjusted *p* < 0.05).

Sex dimorphism has been increasingly identified in host-microbiota interaction^43^. Here, to assess the gender-specificity of microbiota-metabolome signatures in light of the humoral gut-brain axis, we revisited aligned feature tables and performed two-way analysis of variance (ANOVA) for all ion features considering both variables of microbiota (GF/COVN-R) and gender (male/female). For features with significant main effects of microbiota, a considerable proportion also exhibited microbiota×gender interaction in feces (32.6% out of 63.0%, i.e., 51.7%, HESI+), while less for sera (36.5%, HESI+) and least for the cortical brain (10%, HESI+) (adjusted *p* < 0.05) (Fig. 8a); for a more general check, a main effect of gender has been identified for a number of features in all three matrices, among which fecal features embraced the largest proportion with microbiota×gender interaction (82.8%, HESI+), while less for sera (51.3%, HESI+) and least for brain tissues (11.0%, HESI+) (Fig. 8b). Grouping data by gender, post hoc Tukey’s honestly significant difference (HSD) test was conducted comparing GF and CONV-R (adjusted *p* < 0.05) to examine whether individual features of significant main effects of microbiota exhibited sex-specificity (i.e., only of GF/CONV-R difference either in male or female) with the gender-specific distribution summarized in Venn diagrams (Supplementary Fig. 4) and pie charts (Fig. 8c–e). In parallel, we revisited the annotation tables to examine gender specificity of the altered metabolites, yielding PieDonut distributions (Fig. 8f–h) that embraced a consistent trend with ion feature pie chart (i.e., feces < sera ≈ brain) in terms of the proportion of sex-specific molecular signatures. Beyond GF/CONV-R comparison and for a more general check, we also performed Tukey’s HSD test on GF/CONV-R grouped data to compare separately for male and female, with ion feature distribution summarized in Venn diagrams and pie charts (Supplementary Fig. 5).

To examine sex-specific biochemical details, MetaMapp network graphs (Supplementary Fig. 6–8) and MSEA charts (Supplementary Fig. 9) were generated from sex-specific metabolites of GF/CONV-R difference (Supplementary Data 11). We noted for fecal metabolomes amino acids, oligopeptides, oxylipins, bile acids, and nucleobases that were either only altered in males (67 compounds) or in females (60 compounds) (Supplementary Fig. 6). Specifically, tryptophan catabolites kynurenate, xanthurenate, indole-3-carboxaldehyde, and 4-(2-aminophenyl)-2,4-dioxobutanoate were elevated exclusively in male CONV-R feces compared to GF males (Supplementary Fig. 6a). Known microbial products also exhibited alterations selective to gender, as represented by sebacate and adipate only for male feces (Supplementary Fig. 6a) and by 2,3-butadione, a known bacterial toxin linked to health conditions, such as inflammatory bowel diseases^44^, cystic fibrosis^45^, and nonalcoholic fatty liver disease^46^ that was enriched only in the female fecal metabolome when microbiota was present (Supplementary Fig. 6b). For sera, we observed a realm of lipids, organic acids, and amino acids that exhibited gender-specific qualities (Supplementary Fig. 7). Specifically, for male blood sera, multiple acylcarnitines had differentiated levels between GF and CONV-R (Supplementary Fig. 7a), while female sera involved an array of phospholipids, oxylipins, and long-chain fatty acids that were all lower in CONV-R than in GF (Supplementary Fig. 7b). We also noted several aromatic amino acid derivatives that were selectively altered in females but not in males, including tryptophan catabolites (e.g., indole-3-carboxaldehyde, indole-3-lactate) and ferulate (a neuroprotectant)^47^. As for metabolites in the cortical brain, we discovered 13 altered specific to male mice and nine to females, with multiple compounds involved in redox homeostasis and energy metabolism (Supplementary Fig. 8). Of note, prostaglandins (B2 and E2), GSH, AKG, and nicotinamide were only altered in male cortical brains (Supplementary Fig. 8a), while SAH, indole-3-lactate, caproate, 2-hydroxybutyrate, and eicosapentaenoate (EPA) underwent changes only in females (Supplementary Fig. 8b). Taken together, the microbiota-metabolome-brain axis exhibited gender-specific characteristics that need to be addressed in future studies of microbiota’s functional roles in the given pathophenotypes.

## Discussion

Recent data support that commensal microbiota regulates mammalian brain function, opening doors of targeting microbiota for neuroprotective causes. However, biochemical underpinnings for such microbial effects in light of the gut-brain axis remain largely unclear. Metabolomics can address this but often encounters low compound identification rates due to limited references and tools, such as commercially available chemical standards and mass spectral databases. To expand metabolome coverages, recent upgrade of accurate-mass measurement and cheminformatic tools shows promise for enabling high-throughput in silico prediction of elemental composition, molecular structures, and chromatographic retention time with integrated and validated procedures. In this light, we sought to apply a complete suite of cheminformatic rules and tools (untargeted annotation procedures), alongside the conventional use of an in-house library based on hundreds of reference chemical standards (targeted annotation procedures), to conduct high coverage mapping for the biochemical landscape of the “microbiota-gut-brain axis.” Fecal matter, blood sera, and cerebral cortical brain tissues of GF and CONV-R C57BL/6 mice of 8-week-old were harvested, extracted, and analyzed using high-resolution LC-HESI-HRMS. Using statistics and cheminformatics, we screened 20,939 distinct ion features combining both HESI modes and identified hundreds of altered metabolites, including 533 for fecal, 231 for serum, and 58 for brain tissues. To our knowledge, these datasets represent the first high-coverage metabolome characterization for microbiota’s effects on host biochemistry in light of humoral routes for interorgan transport and gut-brain communication.

We gained a “landscape” view of these microbiota-mediated biochemical changes through both the chemical and pathway enrichment analyses. For metabolism in gut lumen where trillions of microbes reside and constant host-microbiota interaction occurs and converges, a total of 70 chemical class clusters and 71 pathways were significantly perturbed, represented by 14 amino acid pathways (aromatic amino acids in particular), purines, bile acids, porphyrins, vitamins (e.g., B and K), and polyamines alongside massive protein catabolism (over 100 oligopeptides altered) and energy harvest events (e.g., TCA cycle, acylcarnitine shuttle). In compliance with past studies^22–24^, fecal aromatic amino acids, especially the tryptophan and tyrosine metabolism, stood out with high statistical significance and in the 50 multiple top-ranked metabolite members in random forest classification. We offered an integrated view of fecal aromatic amino acid changes and observed for microbiota-harboring mice (i) enhanced fluxes of tryptophan pathways featuring decreased tryptophan and enriched indoles, serotonin, xanthurenate, and kynurenate, (ii) altered shikimate pathway with lowered shikimate and increased levels of chorismate derivatives, (iii) suppressed folate biosynthesis, and (iv) enriched tyrosine and downstream by-products including catecholamine neurotransmitters. Of significance, we also identified over 30 altered fecal bile acids with the free-form bile acids (primary and secondary) much elevated while conjugated primary bile acids significantly lowered in the presence of microbiota, including those associated with neurodegenerative disease outcomes, such as Alzheimer’s disease^11,48^.

Systematic effects of microbiota on blood circulation were characterized, as represented by 231 altered metabolites of 27 chemical classes and 61 significantly perturbed pathways spanning porphyrin metabolism, ketone bodies, bile acid biosynthesis, and a range of amino acids, etc. Note that many of the serum pathways were also perturbed in the gut, with the latter generally embracing a larger number of altered metabolites in the given pathway in parallel with compartment-specific details to note. For instance, similar to intestinal changes, microbiota-harboring mice exhibited distinct serum aromatic amino acids featuring (i) enriched levels of microbiota-derived indoles such as indoxyl sulfate and IPA (an important ligand to Pregnane X receptors^49,50^), (ii) altered shikimate pathway with decreased levels in shikimate-3-phosphate and quinate, and (iii) enriched phenolic metabolites phenyllactae, hippurate, salicylate, and phenyl sulfate, while no differences were detected for tyrosine and associated catecholamine products as observed in the gut. Notably, the shikimate pathway, which mammalian cells lack but heavily involved in microorganisms, were detected to be perturbed by microbiota with effects on other aromatic amino acids and associated neurotransmitter generation (e.g., serotonin, tyramine). This suggests a need to evaluate the microbiota-shikimate-host network and associated health risks, for example, of using shikimate pathway-inhibiting neurotoxic herbicides (e.g., glyphosate) that were long believed to be innocuous for mammals^51^. On the molecular level, for instance, random forest classification ranked 4-ethylphenol, a microbial metabolite of tyrosine^29,30^ and the precursor of the microbial 4-ethylphenylsulfate^6^, as a top metabolite variable to CONV-R/GF group separation. Together, these microbiota-driven systemic changes coupling to gut metabolic alteration may serve as a valuable reference for future studies probing microbial influences on host physiology and health, warranting efforts to further delineate genes, enzymes, and transporters involved as well as specific microbial members in play.

We also applied high-coverage metabolomics searching for brain signatures owing to microbiota (Supplementary Fig. 1). Targeting the cerebral cortical brain region, we sought to find proof-of-principle of the microbiota-brain network. In total, 58 altered brain metabolites were identified, spanning oxidative stress makers from methionine/glutathione metabolites, eicosanoid prostaglandins, ketone bodies, to microbiota-derived metabolites such as butyrate, indoxyl sulfate, TMAO, and shikimate/phenylalanine derivatives hydroxyphenyllactate and PAG. Overall, the results were consistent with recently emerging studies, featuring specific metabolites (e.g., 2-hydroxybutyrate, TMAO, glutathione, and acetylneuraminate)^13,52^ and pathways (e.g., oxidative stress, transsulfuration)^13,53,54^, while complementing well with others that touched upon short-chain fatty acids^55^, microbe-associated molecular patterns (e.g., lipopolysaccharides)^56^ and phenolics^57^. Our new data support microbiota’s control over physiological conditions of the CNS, particularly in transsulfuration, redox homeostasis, and neuroinflammation.

One central theme of the microbiome field is to probe the neuromodulatory activities of resident microbiota in light of gut-brain signaling, given their large capacity to produce neurotransmitters and associations with many mental and/or neurologic disease outcomes such as depression^7^, autism spectrum disorder^6,58^, Alzheimer’s disease^48^, and Parkinson’s disease^10^. However, only a few molecular cues underlying such microbiota-gut-brain axis have been reported to date, leaving the mechanistic underpinnings largely unclear^13^. Our high-coverage approach incorporating targeted and untargeted annotation procedures allowed us to address this; the metabolomes of GF/CONV-R difference, either with common or opposite trends among sample matrices, indicate possibilities of direct or indirect humoral pathways, as partially supported by reported knowledge concerning interorgan transport of gut microbial metabolites and in turn, their effects on permeabilities of the BBB^22,59,60^.

We discovered for gut microbiota as a master regulator of neurotransmitter production in the GI tract, as demonstrated in (i) massive depletion of over 15 “classical” neurotransmitters without microbiota, (ii) integrated mapping of changes in catecholamine biosynthesis and glutamine/glutamate-GABA metabolism, as well as (iii) tryptophan pathway-derived neurotransmitters (e.g., serotonin, kynurenate). Further, we found extensive control of microbiota over their transformation as well, particularly through deconjugation. The results complement well recent studies confirming gut bacteria as neurotransmitter producers while supporting local gut microbial capacities to reactivate phase II metabolites from their conjugated forms (e.g., glucuronides, sulfates), likely through hydrolytic enzymes such as β-glucuronidases (GUSs) and/or β-glucosidases^61,62^. Interestingly, except for histamine, glycine, aminoadipate, and cortisol, the majority of neurotransmitters with distinct fecal patterns were either not detected, exhibited no statistical difference, or showed opposite trends (e.g., GABA, histamine) in the serum metabolome; of note, serum serotonin levels were significantly elevated in male CONV-R mice (compared with male GF) but were not significantly different between female groups. These together show that despite the large metabolic activities of the microbe-neurotransmitter network in the intestine, humoral transport and/or systemic effects of these microbiota-derived neurotransmitters are relatively confined in gut and can be affected by gender. Such stringent organ compartmentation and gender-specific characteristics of microbial neurotransmitters need to be studied in the future. That said, we nevertheless discovered a range of compounds potentially involved in interorgan transport and gut-brain communication, for which the presence of microbiota contributed surging levels extending from gut and/or blood to the cerebral cortex. These spanned indoles (e.g., indoxyl sulfate, indole-3-lactate), phenolics (e.g., 4-ethylphenol, phenyl sulfate, PAG), choline derivatives (e.g., TMAO), vitamin B (e.g., pantothenate), butyrate, and methionine, with some virtually absent in GF mice. Of note, indoxyl sulfate, a known uremic toxin and an oxidative stress marker originated from gut bacterial indoxyl^63,64^, embraced the highest fold changes of elevation in both circulating blood (4,351.6-fold) and brain tissues (26.8-fold) with the presence of microbiota. Likewise, TMAO was ranked the third for highest fold changes in both serum (267.3-fold) and brain (7.32-fold) alongside elevated levels in gut lumen (5.90-fold) owing to microbiota. TMAO, also a uremic toxin and an oxidative stress marker, is an oxidation product of gut bacterial trimethylamine through host hepatic flavin monooxygenase with demonstrated adverse effects in cardiovascular diseases^65^. On the contrary, antioxidant agents such as pantothenate and *Bifidobacterium longum* product indole-3-lactate^66^, were also much elevated in both serum and brain when microbiota was present. Together, these findings buttress the notion that commensal microbiota can mediate the CNS (especially redox homeostasis and neuroinflammation) through direct humoral pathways, but the effects may vary depending on multiple factors such as specific microbiota composition and their metabolites, as well as gender, age, and the health status of mammalian hosts^29^.

The strength of this study lies in the use of germ-free mouse model, the sensitive and high-coverage metabolomics approach, and a suite of statistics and visualization tools that allows complete profiling and mining of microbiota-specific molecular signatures as well as their multicompartmental comparison for microbiota-gut-brain interpretation. Cautions and limitations of the present study include: (i) the metabolomics results are by nature descriptive, necessitating more focused efforts testing specific hypotheses of humoral interorgan connections through, for example, fecal transplantation experiments and stable isotope tracer-assisted flux analysis of specific pathways in question; (ii) the metabolome changes observed in this study should be considered as molecular phenotypic representations of host-microbiota interaction rather than microbial effects alone^35^; (iii) use of peak area for statistics instead of absolute molar concentrations essential for inferring actual metabolic activities at given organ matrices; (iv) level 2 annotations confirmed with accurate-mass MS/MS analyses and machine learning-predicted retention time in this study may still fall short for an unambiguous stereoisomeric assignment; (v) biochemical pathways were interpreted for breadth and mostly from a reductionist angle, warranting studies to further delineate at bacteria- and/or pathway-specific levels and to investigate interaction between seemingly distinct chemical classes. In summary, we present here a high-coverage metabolomics approach for characterizing microbiota’s effects on host metabolome in light of interorgan transport and gut-brain crosstalk in mice; the novel findings and insights from this study may prove valuable for future microbiome research.

## Methods

### Chemicals and reagents

LC/MS-grade (Optima^TM^) solvents including methanol (MeOH; catalog #A456-4), acetonitrile (ACN; catalog #A955-4), water (catalog #W6-4) and formic acid (catalog #A117-50), were obtained from Fisher Scientific (Waltham, WA, USA). Stable-isotope-labeling internal standards for a range of classical neurotransmitters and tryptophan catabolites, including acetylcholine-d13 (N,N,N-trimethyl-d9; 1,1,2,2-d4) (d13-ACh; catalog #D-1780), γ-aminobutyric acid-d2 (d2-GABA; catalog #D-1731), L-glutamine-2,3,3,4,4-d5 (d5-Gln; catalog #D-2532), L-tryptophan-2,3,3-d3 (d3-Trp; catalog #D-7419), serotonin-α,α,β,β-d4 (d4-5HT; catalog #D-1550), L-kynurenine (ring-d4, 3,3-d2) (d6-Kyn; catalog #DLM-7842), indole-3-acetic-2,2-d2 acid (d2-IAA; catalog #D-1709) and indole-3-propionic-2,2-d2 acid (d2-IPA; catalog #D-7686) were obtained from CDN Isotopes (Pointe-Claire, Quebec, Canada) and Cambridge Isotopes (Tewksbury, MA, USA). For quality control, these internal standards were spiked in sample pretreatment for monitoring sample recovery and analytical variability.

### Animals

All animal procedures were approved by the Institutional Animal Care & Use Committee (IACUC) at The University of North Carolina at Chapel Hill (UNC) under study protocol number 19-235.0. Conventionally raised (CONV-R) wild-type C57BL/6 mice were obtained from the Jackson Laboratory (Bar harbor, ME, USA) and housed under specific-pathogen-free (SPF) conditions at the UNC animal facility for multiple generations; germ-free (GF) mice were generated and housed in stringently germ-free chambers for generations at the National Gnotobiotic Rodent Resource Center of UNC in Association for Assessment and Accreditation of Laboratory Animal Care International (AAALAC)-accredited facilities (Chapel Hill, NC, USA). C57BL/6 mouse littermates of ~7 weeks old raised under GF and conventional conditions were age-matched upon selection for sacrifice and analyses, resulting in a final total of 24 mice, including 12 GF mice and 12 SPF CONV-R mice with 6 males and 6 females included in each. The animals were raised under the following conditions: 22 °C, 40–70% humidity, and a daily 12:12 h light-dark cycle. For dietary administration, we consistently fed all mice the same purified (and sterile) standard Prolab RHM 3000 pelleted rodent diets (St. Louis, MO, USA) and provided tap water ad libitum. Prior to sacrifice and sample harvest, all mice were observed under their original housing conditions for one week; animals were not considered if they exhibited significant signs of serious injury or morbidity (e.g., malocclusion or fight wounds). Upon euthanization in CO_2_ chambers, feces, brain, and blood sera were harvested, snap-frozen, and stored in -80 °C freezer before analysis.

### Sample extraction

Fecal matter, blood sera, and brain tissues (of the cerebral cortical region) were aliquoted and extracted for metabolome analyses. To maximize coverage and throughput, extraction procedures were kept as simple and nonselective as can be. For feces, ~20 mg thawed samples were aliquoted to a 1.5-mL Eppendorf tube (Hamburg, Germany) with ~30 mg acid-washed glass beads added (Sigma-Aldrich, St. Louis, MO). For every 10 mg feces aliquots, 300 μL of ice-cold MeOH:water (50:50, *v/v*) with spiked ISDs were added for extraction. The samples were homogenized on a TissueLyzer (Qiagen, Hilden, Germany) at 50 Hz for 10 min and centrifuged at 12,000 × *g* for 10 min (Eppendorf, Hamburg, Germany). A 100-μL aliquot of the supernatant was transferred and dried in a CentriVap vacuum evaporator (Labconco, MO, USA). For serum, 20 μL was aliquoted from each sample in a 1.5-mL Eppendorf tube (Hamburg, Germany). A total of 180 μL ice-cold ISD-containing MeOH was added, vortexed, and incubated at -20 °C freezer for 30 min. The extracts were centrifuged at 15,000 × *g* for 10 min for precipitation of particulates and proteins. Aliquoted 100-μL of supernatants were SpeedVac-dried as described above. For cerebral cortical brain tissues, ~20 mg was aliquoted from each sample to a 2-mL screw cap microcentrifuge tubes (VWR, Radnor, PA, USA) that contained clean stainless-steel beads of 5-mm i.d. To every 20 mg tissue, 400 μL of the ice-cold ISD-containing MeOH was added for extraction. The samples were homogenized on a TissueLyzer (Qiagen, Hilden, Germany) at 50 Hz for 2 min and further incubated at -20 °C for 1 h prior to centrifugation at 18,000 × *g* for 10 min. One hundred-μL of the extractant supernatants were SpeedVac-dried as described before. Upon instrumental analysis, all dried extracts, including feces, sera, and brain tissue, were resuspended in 50 μL ACN:water (2:98, *v/v*) for injection.

### Instrumental analysis

Global metabolome profiling was performed on a Thermo Fisher Scientific Vanquish UHPLC coupling to a high-resolution accurate-mass (HRAM) Q-Exactive mass spectrometer interfacing with a heated electrospray ionization (HESI) source and a hybrid quadrupole-orbitrap mass analyzer (Waltham, MA, USA). For chromatographic separation, a Waters Acquity UPLC HSS T3 column (100Å, 1.8 μm, 2.1 mm × 100 mm) was used (Milford, MA, USA). The mobile phases consisted of 0.1% formic acid in water (A) and 0.1% formic acid in ACN (B), flowing at a rate of 0.4 mL/min under 40 °C with a 15-min gradient: 2% B, 0–1 min; 2 to 15% B, 1–3 min; 15 to 50% B, 3–6 min; 50 to 98% B, 6–7.5 min; 98% B, 7.5–11.5 min; 98 to 2% B, 11.5–11.6 min; 2% B, 11.6–15 min. HESI positive and negative ionization were both performed, with following ion source setting: sheath gas 60 L/min, aux gas 10 L/min, sweep gas 1 L/min, spray voltage 2.75 kV, capillary 325 °C, and aux gas heater 400 °C. All mass spectral data (in *.RAW format) were acquired through the Thermo XCalibur program (version 4.1) (Waltham, MA, USA). For MS1 full scan profiling (Q not used) aiming at examining global metabolomic profiles while screening for distinct ion features, the mass spectrometer was operated scanning mass range of 80-1,000 Da with a mass resolution of 70,000 full width at half height (FWHM) at 200 Da. For MS/MS spectra acquisition in later unknown structural annotation stages (with both Q and orbitrap running), the mass spectrometer was operated scanning for 80-1,000 Da in full-scan MS1 mode (FWHM 70,000; AGC target 3e6; max IT 200 ms) alternating with parallel reaction monitoring (PRM) MS/MS mode with normalized collision energy averaging 10, 50 and 100 (FWHM 17 500; AGC target 2e5; max IT 50 ms).

### Data processing, statistics, and visualization

MS1 full-scan *.RAW data were converted to *.mzXML files in ProteoWizards MS Convert (Palo Alto, CA, USA) and uploaded to Scripps XCMS^67^ (La Jolla, CA, USA) for data processing, resulting in master peak tables aligning all samples separately from three sample types acquired under two HESI polarity modes. The dataset was analyzed in R by Welch’s *t*-test with adjusted p-values (i.e., *q* values) calculated based on the Benjamin-Hochberg procedure to compare GF and CONV-R groups. To further determine the effect of gender, two-way analysis of variance (ANOVA) was conducted. Raw peak areas for each ion feature were pareto-scaled and log-transformed to meet the test assumptions of ANOVA (e.g., no significant outliers, normality, and homogeneity of variance). Then, two-way ANOVA and post hoc Tukey’s (honestly significant difference) HSD test were performed to determine the main effects of microbiota and gender and to test for within-group statistical difference, respectively. Plots and analyses including Box and Whisker plot, barplot, principal component analysis score and scree plot, variable importance plot, PieDonut chart, as well as heatmaps were generated in R (version 4.0.1) (Vienna, Austria) using packages including readxl (version 1.3.1), dplyr (version 1.0.6), tidyverse (version 1.3.1), ggplot2 (version 3.3.3), ggpubr (version 0.4.0), ggthemes (version 4.2.4), ggsignif (version 0.6.1), RColorBrewer (version 1.1.2), dendextend (version 1.15.1), randomForest (version 4.6.14), FactoMineR (version 2.4), factoextra (version 1.0.7), pheatmap (version 1.0.12), wesanderson (version 0.3.6), and webr (version 0.1.5)^68^. Specifically for principal component analysis, we performed Pareto scaling first and included all ion features from the alignment table for the given sample matrix under specific heated ESI mode; for a showcase, the PCA score plots were generated using all 17,386 ion features for feces (HESI+) (Fig. 2a, b) and 10,012 features in total for blood sera (HESI+) (Fig. 5a, b). Random forest classification was conducted using the randomForest (version 4.6.14) and rfPermute (version 2.2) packages of R (Vienna, Austria) to generate variable importance plots to rank individual metabolites, with hyperparameters and permutation results provided (Supplementary Information)^69^. Metabolite networks were constructed using a MetaMapp approach^33^ (web-based portal, version 2020) that calculated biological pathways relevance (KEGG reactant pairs) and chemical structural similarity (Tanimoto coefficient > 0.7) which were further visualized in CytoScape with version 2.7.2 for Window 10 OS and version 3.8.0 for macOS (Seattle, WA, USA). Chemical similarity enrichment analysis (ChemRICH) plots were constructed to visualize changing metabolites based on chemical classes (Davis, CA, USA)^19^. Quantitative metabolite set enrichment analysis (qMSEA) was conducted on altered metabolites in MetaboAnalyst 4.0 (Montreal, QC, Canada) using their PubChem CID identifiers and log-transformed peak area pairs as data input to compute based on 99 a priori defined sets of metabolites in pathway-associated metabolites sets (SMPDB); metabolite sets that contained at least two compounds were used.

### Compound identification

Since the millennium, untargeted/global metabolomics has been fast evolving with significant development in gas/liquid chromatography-mass spectrometry technologies, featuring the rise of accurate-mass measurement that has since opened doors of confident de novo determination of elemental composition and chemical structure in a high-throughput, sensitive, and nonselective manner^70^. The conceptual differences between the untargeted analysis, targeted analysis, and the like in metabolomics necessitates clear elucidation. The untargeted/global metabolomics analysis aims to detect as many features as possible in a single run and after a differential statistical comparison, the significant ones are sent to compound identification for hypothesis generation; annotation strategies can be either knowledge-based/targeted (matching against a predetermined in-house spectral library or experimental databases) or data-driven/untargeted (inferring directly from data, e.g., using streamlined in silico cheminformatic algorithms and scoring). While, in the case of targeted metabolomics, one has previous information of what he/she will look for, such as precursor/fragment transitions, and usually seeks a quantitative analysis to determine the absolute concentration levels using commercial labeled standards. In this study, we conducted global full-scan profiling of all ion features, performed statistics for those aligned across all samples, and leveraged both targeted (with known reference) and untargeted (infer from data directly) cheminformatic strategies for annotation with goals to identify/annotate all ion features with statistical significance (Supplementary Fig. 1). We refer to our approach as “high-coverage annotation,” since both the knowledge-based/targeted and data-driven/untargeted annotation strategies were performed on all significant ion features in a streamlined manner, allowing for integrated scoring, cross-validation, and postretention time prediction and filtering that helped achieve highest metabolome coverage possible.

Specifically, we collected a total of 20,939 tandem mass spectra of distinct ion features (pertaining to the microbiota-gut-brain connection) for structural annotation. These included 16,001 for feces, 3,977 for sera, and 961 for cerebral tissues combining HESI positive and negative modes. The *.RAW data were converted into *.abf format using the Reifycs Abf Converter (https://www.reifycs.com/AbfConverter/), uploaded to MS-DIAL 4.16 (Riken, Japan)^71^ to obtain *.MAT files that contained MS1 accurate mass, MS1 isotopic abundances, MS/MS spectra, and retention time for each ion feature with an acquired tandem mass spectrum. The targeted annotation procedure exploits an in-house experimental library established from 423 authentic chemical standards of canonical pathways, neurotransmitters, and literature-reported microbial metabolites; the local library stores chromatographic retention time, accurate mass, and characteristic MS2 fragment ions. For untargeted analyses, a cutting-edge cheminformatic pipeline was used integrating machine learning-based retention time predication (Retip)^16^, embedded experimental mass spectral database search (GNPS^72^, MassBank^73^, and ReSpect^74^, totaling 28,293 spectra), and rule-based in silico cheminformatic analysis (MS-FINDER, version 3.30)^15^ which integrates a number of cheminformatic algorithms while querying major biomolecule chemical databases, such as HMDB, LipidMAPS and PubChem Biomolecules adding up to over 100 million structures. The detailed settings of MS-FINDER are provided in Supplementary Information (Page 12). Importantly, the elemental composition was predicted (based on MS1 accurate mass, MS1 isotopic ratios, and MS/MS product ions) and filtered based on the Seven Golden Rules^14^, which refers to an algorithm consisting of seven heuristic rules, i.e., (i) restriction for element numbers, (ii) LEWIS and SENIOR rules, (iii) isotopic patterns, (iv) H/C ratios, (v) element ratios of N, O, P, S vs. C, (vi) elemental ratio probabilities, and (vii) presence of trimethylsilylation (for GC/derivatization platforms if applicable). Afterwards, the top five candidates were sent to structural dereplication, and chromatographic retention times were predicted afterward for these structures using the Retip (version 0.5.4) and h2o (version 3.32.1.3) package of R (Vienna, Austria) and compared with true values for lipophilicity validation and annotation cleanup. The resultant top-score structures were manually assessed and curated carefully to boost annotation confidence; the postcuration strategies included, but not limited to, chemical inference, biological relevance, comparison of targeted and untargeted results, and the use of additional experimental mass spectral database search including Metlin (La Jolla, CA, USA), MoNA (Davis, CA, USA), HMDB (Edmonton, AB, Canada), and mzCloud (Waltham, MA, USA).

The application of specific quality criteria to filter out low-quality MS/MS data and annotations is not only common but essential in untargeted metabolomics practices. In this study, however, we did not exclude any ion features of low abundance (under MS1 full-scan) for MS/MS acquisition, nor did we apply thresholds of minimum/noise of ion intensity for filtering out low-quality/noisy tandem MS/MS. This is because (i) we aimed for untargeted annotation in an unbiased manner for all ion features regardless of low abundances, and (ii) our integrated and streamlined scoring strategy carried a penalty mechanism itself (Supplementary Fig. 1c), meaning, if the tandem mass spectrum per se is too noisy or the quality is poor, the resultant scores will be correspondingly low, and the annotations will be naturally filtered out from the final annotation list (Supplementary Fig. 1d). For the finalized tables of the changed fecal metabolome (Supplementary Data 2), serum metabolome (Supplementary Data 3), and brain metabolome (Supplementary Data 4), we only included identification/annotation results of highest confidence, i.e. level 1 confirmed structure and level 2 probable structure according to the Metabolomics Standard Initiative (MSI) to best inform future studies on microbiota in health and diseases^17,75,76^.

## Supplementary information

Supplementary Information

Description of Additional Supplementary Files

Supplementary Data 1

Supplementary Data 2

Supplementary Data 3

Supplementary Data 4

Supplementary Data 5

Supplementary Data 6

Supplementary Data 7

Supplementary Data 8

Supplementary Data 9

Supplementary Data 10

Supplementary Data 11

## Data Availability

The relevant metabolomics raw data and master alignment ion feature tables generated for this study have been deposited in the National Metabolomics Data Repository (accession IDs: ST001756, ST001757, and ST001758) with Project No. PR001126 (10.21228/M8569X). All source data for figures and tables of this work are available online at Zenodo (10.5281/zenodo.5016278) and/or GitHub (https://github.com/darciliz/nc_hcm_code); requests for additional information or data can be addressed to the corresponding author. Experimental and in silico mass spectral databases used for postcuration/validation purposes included MassBank of North America (MoNA) (https://mona.fiehnlab.ucdavis.edu/), Metlin (https://metlin.scripps.edu/), HMDB (https://hmdb.ca/), and mzCloud (https://www.mzcloud.org/). Source data are provided with this paper.

## Source data

Source Data

## Code availability

R Codes and associated data for reproducing part of the figures in this study have been deposited at GitHub (https://github.com/darciliz/nc_hcm_code). Unless noted otherwise, all other analyses including ChemRICH and MetaMapp were done using web-based portal and visualized in standalone GUI software such as Adobe Illustrator (version 2020; 2021) and CytoScape (version 2.7.2 for Window 10 OS and version 3.8.0 for macOS).

## References

[1] Turnbaugh PJ (2007). The human microbiome project. Nature.

[2] Turnbaugh PJ (2006). An obesity-associated gut microbiome with increased capacity for energy harvest. Nature.

[3] Hooper LV, Littman DR, Macpherson AJ (2012). Interactions between the microbiota and the immune system. Science.

[4] Wells JM, Rossi O, Meijerink M, van Baarlen P (2011). Epithelial crosstalk at the microbiota–mucosal interface. Proc. Natl Acad. Sci..

[5] Cryan JF, O’Riordan KJ, Sandhu K, Peterson V, Dinan TG (2020). The gut microbiome in neurological disorders. Lancet Neurol..

[6] Hsiao ElaineY (2013). Microbiota modulate behavioral and physiological abnormalities associated with neurodevelopmental disorders. Cell.

[7] Valles-Colomer M (2019). The neuroactive potential of the human gut microbiota in quality of life and depression. Nat. Microbiol.

[8] de la Fuente-Nunez C, Meneguetti BT, Franco OL, Lu TK (2018). Neuromicrobiology: How microbes influence the brain. ACS Chem. Neurosci..

[9] Needham BD, Kaddurah-Daouk R, Mazmanian SK (2020). Gut microbial molecules in behavioural and neurodegenerative conditions. Nat. Rev. Neurosci..

[10] Kim S (2019). Transneuronal propagation of pathologic α-synuclein from the gut to the brain models Parkinson’s disease. Neuron.

[11] MahmoudianDehkordi S (2019). Altered bile acid profile associates with cognitive impairment in Alzheimer’s disease-An emerging role for gut microbiome. Alzheimers Dement.

[12] Luan H, Wang X, Cai Z (2019). Mass spectrometry-based metabolomics: targeting the crosstalk between gut microbiota and brain in neurodegenerative disorders. Mass Spectrom. Rev..

[13] Konjevod M (2021). Metabolomics analysis of microbiota-gut-brain axis in neurodegenerative and psychiatric diseases. J. Pharm. Biomed. Anal..

[14] Kind T, Fiehn O (2007). Seven Golden Rules for heuristic filtering of molecular formulas obtained by accurate mass spectrometry. BMC Bioinforma..

[15] Tsugawa H (2016). Hydrogen rearrangement rules: computational MS/MS fragmentation and structure elucidation using MS-FINDER software. Anal. Chem..

[16] Bonini P, Kind T, Tsugawa H, Barupal DK, Fiehn O (2020). Retip: retention time prediction for compound annotation in untargeted metabolomics. Anal. Chem..

[17] Schymanski EL (2014). Identifying small molecules via high resolution mass spectrometry: communicating confidence. Environ. Sci. Technol..

[18] Powell N, Walker MM, Talley NJ (2017). The mucosal immune system: master regulator of bidirectional gut–brain communications. Nat. Rev. Gastroenterol. Hepatol..

[19] Barupal DK, Fiehn O (2017). Chemical similarity enrichment analysis (ChemRICH) as alternative to biochemical pathway mapping for metabolomic datasets. Sci. Rep..

[20] Frolkis A (2010). SMPDB: the small molecule pathway database. Nucleic Acids Res.

[21] Chong J (2018). MetaboAnalyst 4.0: towards more transparent and integrative metabolomics analysis. Nucleic Acids Res..

[22] Cervenka, I., Agudelo, L. Z. & Ruas, J. L. Kynurenines: Tryptophan’s metabolites in exercise, inflammation, and mental health. *Science* **357**, eaaf9794 (2017). 10.1126/science.aaf9794 28751584

[23] Platten M, Nollen EAA, Rohrig UF, Fallarino F, Opitz CA (2019). Tryptophan metabolism as a common therapeutic target in cancer, neurodegeneration and beyond. Nat. Rev. Drug Disco..

[24] Dodd D (2017). A gut bacterial pathway metabolizes aromatic amino acids into nine circulating metabolites. Nature.

[25] Strandwitz P (2018). Neurotransmitter modulation by the gut microbiota. Brain Res.

[26] Mittal R (2017). Neurotransmitters: The Critical Modulators Regulating Gut-Brain Axis. J. Cell Physiol..

[27] Volkow ND, Wise RA, Baler R (2017). The dopamine motive system: implications for drug and food addiction. Nat. Rev. Neurosci..

[28] Asano Y (2012). Critical role of gut microbiota in the production of biologically active, free catecholamines in the gut lumen of mice. Am. J. Physiol.-Gastrointest. Liver Physiol..

[29] Nicholson JK (2012). Host-gut microbiota metabolic interactions. Science.

[30] Bone E, Tamm A, Hill M (1976). The production of urinary phenols by gut bacteria and their possible role in the causation of large bowel cancer. Am. J. Clin. Nutr..

[31] Kikuchi K (2019). Gut microbiome-derived phenyl sulfate contributes to albuminuria in diabetic kidney disease. Nat. Commun..

[32] Yano JM (2015). Indigenous bacteria from the gut microbiota regulate host serotonin biosynthesis. Cell.

[33] Barupal DK (2012). MetaMapp: mapping and visualizing metabolomic data by integrating information from biochemical pathways and chemical and mass spectral similarity. BMC Bioinforma..

[34] Karlsson E, Mapelli V, Olsson L (2017). Adipic acid tolerance screening for potential adipic acid production hosts. Microb. Cell Factories.

[35] Visconti A (2019). Interplay between the human gut microbiome and host metabolism. Nat. Commun..

[36] Fernández V, Llinares-Benadero C, Borrell V (2016). Cerebral cortex expansion and folding: what have we learned?. EMBO J..

[37] Friedland RP (2015). Mechanisms of molecular mimicry involving the microbiota in neurodegeneration. J. Alzheimers Dis..

[38] Pandya M, Altinay M, Malone DA, Anand A (2012). Where in the brain is depression?. Curr. Psychiatry Rep..

[39] Hughes AN, Oxford JT (2014). A lipid-rich gestational diet predisposes offspring to nonalcoholic fatty liver disease: a potential sequence of events. Hepat. Med.

[40] Belalcázar AD, Ball JG, Frost LM, Valentovic MA, Wilkinson JT (2014). Transsulfuration is a significant source of sulfur for glutathione production in human mammary epithelial cells. ISRN Biochem.

[41] Chu C (2019). The microbiota regulate neuronal function and fear extinction learning. Nature.

[42] Swann JR, Spitzer SO, Diaz Heijtz R (2020). Developmental signatures of microbiota-derived metabolites in the mouse brain. Metabolites.

[43] Santos-Marcos JA (2019). Sex differences in the gut microbiota as potential determinants of gender predisposition to disease. Mol. Nutr. Food Res.

[44] Ahmed I, Greenwood R, Costello B, Ratcliffe N, Probert CS (2016). Investigation of faecal volatile organic metabolites as novel diagnostic biomarkers in inflammatory bowel disease. Aliment Pharm. Ther..

[45] Whiteson KL (2014). Breath gas metabolites and bacterial metagenomes from cystic fibrosis airways indicate active pH neutral 2,3-butanedione fermentation. ISME J..

[46] Raman M (2013). Fecal microbiome and volatile organic compound metabolome in obese humans with nonalcoholic fatty liver disease. Clin. Gastroenterol. Hepatol..

[47] Liu Z (2019). Ferulic acid increases intestinal Lactobacillus and improves cardiac function in TAC mice. Biomedicine Pharmacother..

[48] Nho K (2019). Altered bile acid profile in mild cognitive impairment and Alzheimer’s disease: Relationship to neuroimaging and CSF biomarkers. Alzheimers Dement.

[49] Alexeev EE (2018). Microbiota-Derived Indole Metabolites Promote Human and Murine Intestinal Homeostasis through Regulation of Interleukin-10 Receptor. Am. J. Pathol..

[50] Venkatesh M (2014). Symbiotic bacterial metabolites regulate gastrointestinal barrier function via the xenobiotic sensor PXR and Toll-like receptor 4. Immunity.

[51] Mesnage, R. et al. Shotgun metagenomics and metabolomics reveal glyphosate alters the gut microbiome of Sprague-Dawley rats by inhibiting the shikimate pathway. *bioRxiv*, 870105, 10.1101/870105 (2019).

[52] Matsumoto M (2013). Cerebral low-molecular metabolites influenced by intestinal microbiota: a pilot study. Front Syst. Neurosci..

[53] Hertel J (2019). Integrated analyses of microbiome and longitudinal metabolome data reveal microbial-host interactions on sulfur metabolism in Parkinson’s disease. Cell Rep..

[54] Luan H (2015). LC-MS-based urinary metabolite signatures in idiopathic Parkinson’s disease. J. Proteome Res.

[55] Erny D (2015). Host microbiota constantly control maturation and function of microglia in the CNS. Nat. Neurosci..

[56] O’Connor J (2009). Lipopolysaccharide-induced depressive-like behavior is mediated by indoleamine 2,3-dioxygenase activation in mice. Mol. Psychiatry.

[57] Valdés L (2015). The relationship between phenolic compounds from diet and microbiota: impact on human health. Food Funct..

[58] De Angelis M (2013). Fecal microbiota and metabolome of children with autism and pervasive developmental disorder not otherwise specified. PLOS ONE.

[59] Braniste V (2014). The gut microbiota influences blood-brain barrier permeability in mice. Sci. Transl. Med..

[60] Angelino, D. et al. 5-(Hydroxyphenyl)-γ-Valerolactone-Sulfate, a Key Microbial Metabolite of Flavan-3-ols, Is Able to Reach the Brain: Evidence from Different in Silico, In Vitro and In Vivo Experimental Models. *Nutrients* **11**, 2678 (2019). 10.3390/nu11112678 PMC6893823 31694297

[61] Bhatt AP, Redinbo MR, Bultman SJ (2017). The role of the microbiome in cancer development and therapy. CA: A Cancer J. Clinicians.

[62] Biernat KA, Li B, Redinbo MR (2018). Microbial Unmasking of Plant Glycosides. mBio.

[63] Wikoff WR (2009). Metabolomics analysis reveals large effects of gut microflora on mammalian blood metabolites. Proc. Natl Acad. Sci..

[64] Evenepoel, P., Meijers, B. K., Bammens, B. R. & Verbeke, K. Uremic toxins originating from colonic microbial metabolism. *Kidney Int Suppl.* **114**, S12–S19 (2009). 10.1038/ki.2009.402 19946322

[65] Wang Z (2011). Gut flora metabolism of phosphatidylcholine promotes cardiovascular disease. Nature.

[66] Meng D (2020). Indole-3-lactic acid, a metabolite of tryptophan, secreted by Bifidobacterium longum subspecies infantis is anti-inflammatory in the immature intestine. Pediatr. Res..

[67] Tautenhahn R, Patti GJ, Rinehart D, Siuzdak G (2012). XCMS online: a web-based platform to process untargeted metabolomic data. Anal. Chem..

[68] R: A language and environment for statistical computing (R Foundation for Statistical Computing, Vienna, Austria, 2010).

[69] Bruce, P., Bruce, A. & Gedeck, P. *Practical Statistics for Data Scientists: 50+ Essential Concepts Using R and Python*. (O’Reilly Media, 2020).

[70] Junot C, Fenaille F, Colsch B, Bécher F (2014). High resolution mass spectrometry based techniques at the crossroads of metabolic pathways. Mass Spectrom. Rev..

[71] Tsugawa H (2015). MS-DIAL: data-independent MS/MS deconvolution for comprehensive metabolome analysis. Nat. Methods.

[72] Wang M (2016). Sharing and community curation of mass spectrometry data with Global Natural Products Social Molecular Networking. Nat. Biotechnol..

[73] Horai H (2010). MassBank: a public repository for sharing mass spectral data for life sciences. J. Mass Spectrom..

[74] Sawada Y (2012). RIKEN tandem mass spectral database (ReSpect) for phytochemicals: a plant-specific MS/MS-based data resource and database. Phytochemistry.

[75] Sumner LW (2007). Proposed minimum reporting standards for chemical analysis Chemical Analysis Working Group (CAWG) Metabolomics Standards Initiative (MSI). Metabolomics.

[76] Schrimpe-Rutledge AC, Codreanu SG, Sherrod SD, McLean JA (2016). Untargeted Metabolomics Strategies-Challenges and Emerging Directions. J. Am. Soc. Mass Spectrom..

