# Supplementary material for: High-coverage metabolomics uncovers microbiota-driven biochemical landscape of interorgan transport and gut-brain communication in mice
Source: Nat Commun. 2021 Oct 19;12:6000. doi: 10.1038/s41467-021-26209-8 (PMC8526691; doi:10.1038/s41467-021-26209-8)
Supplement: Supplementary file 1 — Supplementary Information [file 41467_2021_26209_MOESM1_ESM.pdf]

Supplementary Information:

High-coverage metabolomics uncovers microbiota-driven biochemical landscape of interorgan transport and gut-brain communication in mice

Yunjia Lai †, Chih-Wei Liu †, Yifei Yang †, Yun-Chung Hsiao †, Hongyu Ru †, and Kun Lu †*

† *Department of Environmental Sciences and Engineering, Gillings School of Global Public Health, University of North Carolina, Chapel Hill, NC 27599-7431, United States.*

***Correspondence author**. E-mail: kunlu [at] unc.edu*

List of Items

Supplementary Fig. 1
Schematic illustration of compound identification procedures.

Supplementary Fig. 2
Box and Whisker plots of select serum compounds: indoles and neurotransmitters.

Supplementary Fig. 3
Schematic illustration of altered methionine/glutathione transsulfuration pathway in cerebral cortical brain comparing GF and CONV-R mice.

Supplementary Fig. 4
Distribution of ion features of GF/CONV-R difference considering gender (male/female) as the moderator variable: Venn diagrams for each matrix-HESI combination.

Supplementary Fig. 5
Distribution of ion features of male/female difference considering microbiota (GF/CONV-R) as the moderator variable: Venn diagrams and pie charts.

Supplementary Fig. 6
MetaMapp network of sex-specific metabolites in feces comparing GF and CONV-R mice.

Supplementary Fig. 7
MetaMapp network of sex-specific metabolites in blood sera comparing GF and CONV-R mice.

Supplementary Fig. 8
MetaMapp network of sex-specific metabolites in brain comparing GF and CONV-R mice.

Supplementary Fig. 9
Metabolite set enrichment analysis (MSEA) of gender-specific metabolites.

Additional information
MS-FINDER settings; Random Forest classification for generating VIP plots.

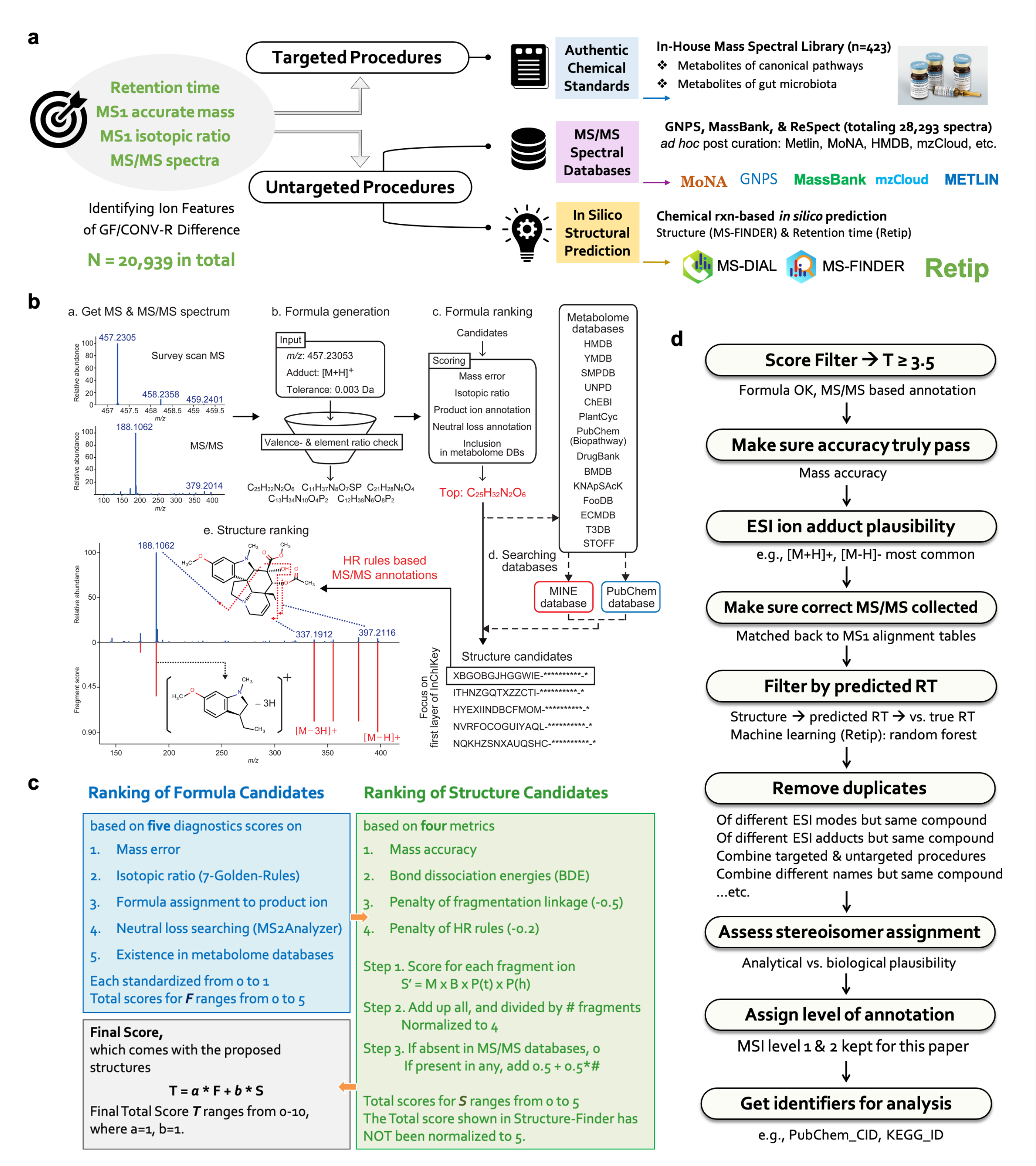

Supplementary Fig. 1. Schematic illustration of compound identification procedures.

(**a**) Workflow combining targeted and untargeted annotation procedures; License-wise, the symbols of lightbulb and target/arrow were purchased from Iconfinder.com (transaction No. 0003685707, 0003685712), symbols of library and database were drawn by the first author of this article using Adobe Illustrator 2021, while the logos of MS-DIAL and MS-FINDER were distributed by CC-BY-SA 4.0 license and could be used for publication purposes; (**b**) *in silico* prediction of chemical formula and structure were based on chemical bond energies, hydrogen-rearrangement rules, and heuristic rules, as detailed in MS-FINDER[1]; (**c**) a streamlined scoring strategy for steps of (**b**); (**d**) strategies and procedures for post-curation of *in silico* annotations in this work.

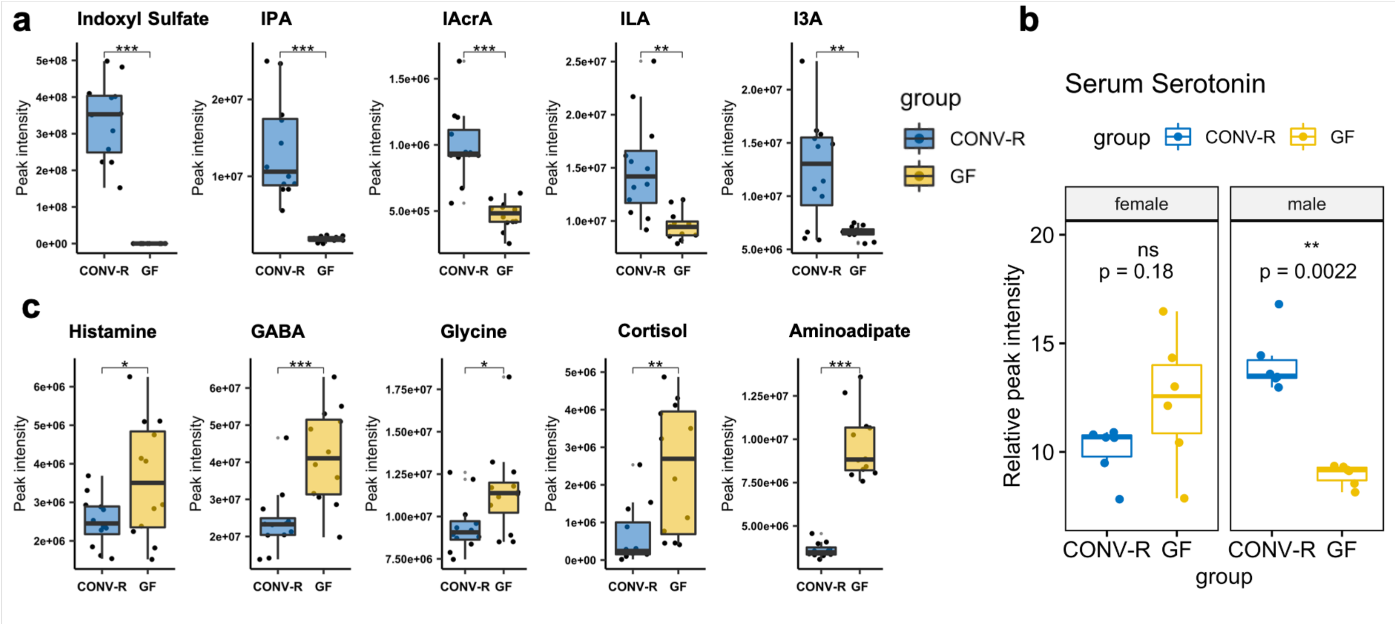

Supplementary Fig. 2. Box and Whisker plots of select serum indoles and neurotransmitters. (**a**) indoles comparing CONV-R (N=12) and GF mice (N=12), with the box ranging from the first quartile to the third while the whiskers going from each quartile to the minimum or maximum, *p<0.05, **p<0.01, ***p<0.001, ****p<0.0001, two-sided Welch's *t*-test; exact p-values and adjusted p-values are provided in Supplementary Table 3; (**b**) serotonin levels examined in a gender-specific manner with the box ranging from the first quartile to the third while the whiskers going from each quartile to the minimum or maximum (N=6 for each group), ns: p≥0.05, *p<0.05, **p<0.01, ***p<0.001, ****p<0.0001, multigroup pairwise Wilcoxon rank-sum test; (**c**) neurotransmitters and neuromodulatory molecules comparing CONV-R (N=12) and GF mice (N=12), with the box ranging from the first quartile to the third while the whiskers going from each quartile to the minimum or maximum, *p<0.05, **p<0.01, ***p<0.001, ****p<0.0001, two-sided Welch's *t*-test; exact p-values and adjusted p-values are provided in Supplementary Table 3.

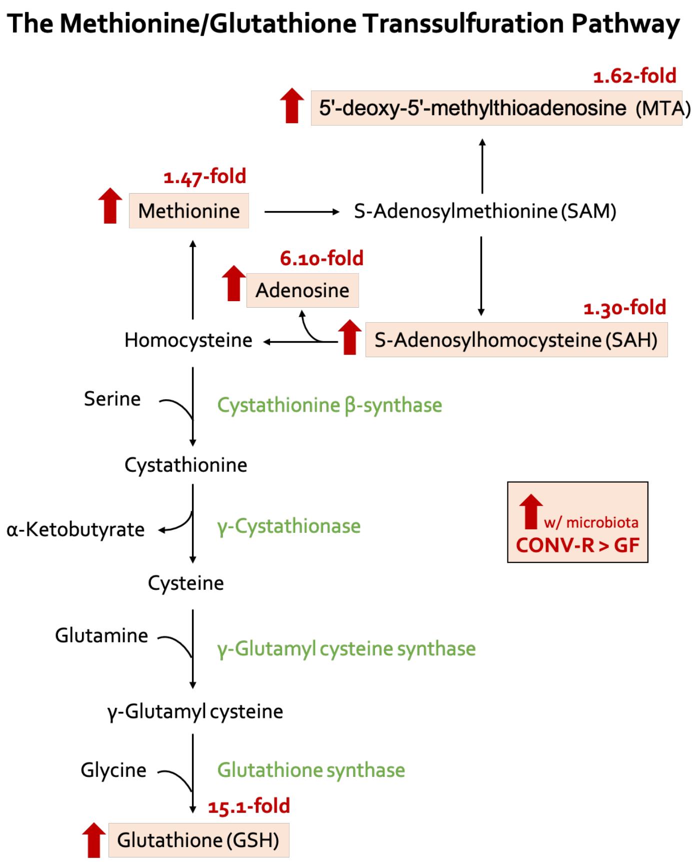

Supplementary Fig. 3. Schematic illustration of altered methionine/glutathione transsulfuration pathway in cerebral cortical brain comparing GF and CONV-R mice[2,3].

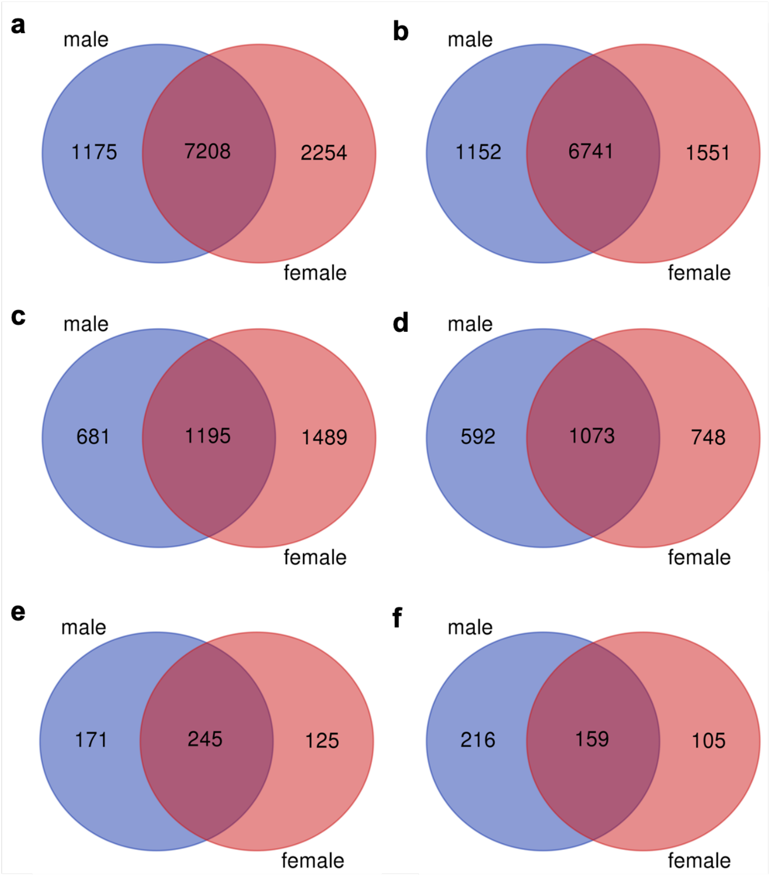

Supplementary Fig. 4. Distribution of ion features of GF/CONV-R difference (focal variable considering gender (male/female, moderator variable) as determined by two-way ANOVA (adjusted p<0.05) and *post hoc* Tukey's HSD test (adjusted p<0.05).

(**a-f**) Venn diagram illustrating ion features of each matrix-HESI combination that had significant main effects of microbiota (two-way ANOVA, adjusted p<0.05) while exhibiting significant GF/CONV-R difference either in male or female or both (Tukey's HSD test, adjusted p<0.05): (a) feces, HESI+; (b) feces, HESI-; (c) serum, HESI+; (d) serum, HESI-; (e) cerebral cortical brain tissues, HESI+; (f) cerebral cortical brain tissues, HESI-.

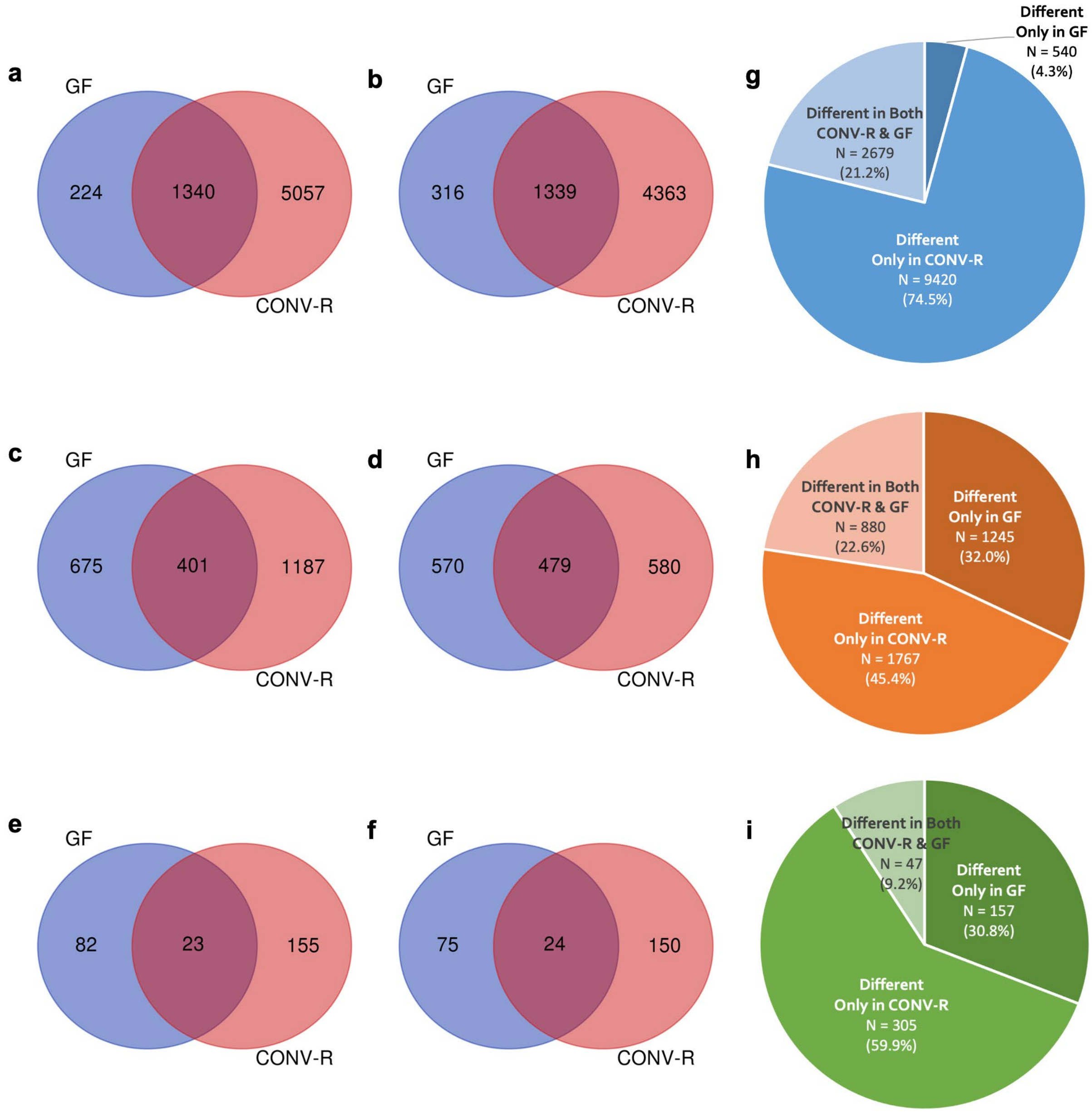

Supplementary Fig. 5. Distribution of ion features of male/female difference (focal variable) considering microbiota (GF/CONV-R) as the moderator variable as determined by two-way ANOVA (adjusted p<0.05) and *post hoc* Tukey's HSD test (adjusted p<0.05).

(**a-f**) Venn diagram illustrating ion features of each matrix-HESI combination that had significant main effects of gender (two-way ANOVA, adjusted p<0.05) while exhibiting significant male/female difference either in GF, CONV-R, or both (Tukey's HSD test, adjusted p<0.05): (a) feces, ESI+; (b) feces, ESI-; (c) serum, ESI+; (d) serum, ESI-; (e) cerebral cortical brain tissues, ESI+; (f) cerebral cortical brain tissues, ESI-. (**g-i**) Pie charts illustrating the distribution of ion features (combining both HESI modes for each sample matrix) that had main effects of gender (adjusted p<0.05) while exhibiting significant male/female difference either in GF, CONV-R, or both based on *post hoc* Tukey's HSD test (adjusted p-value<0.05).

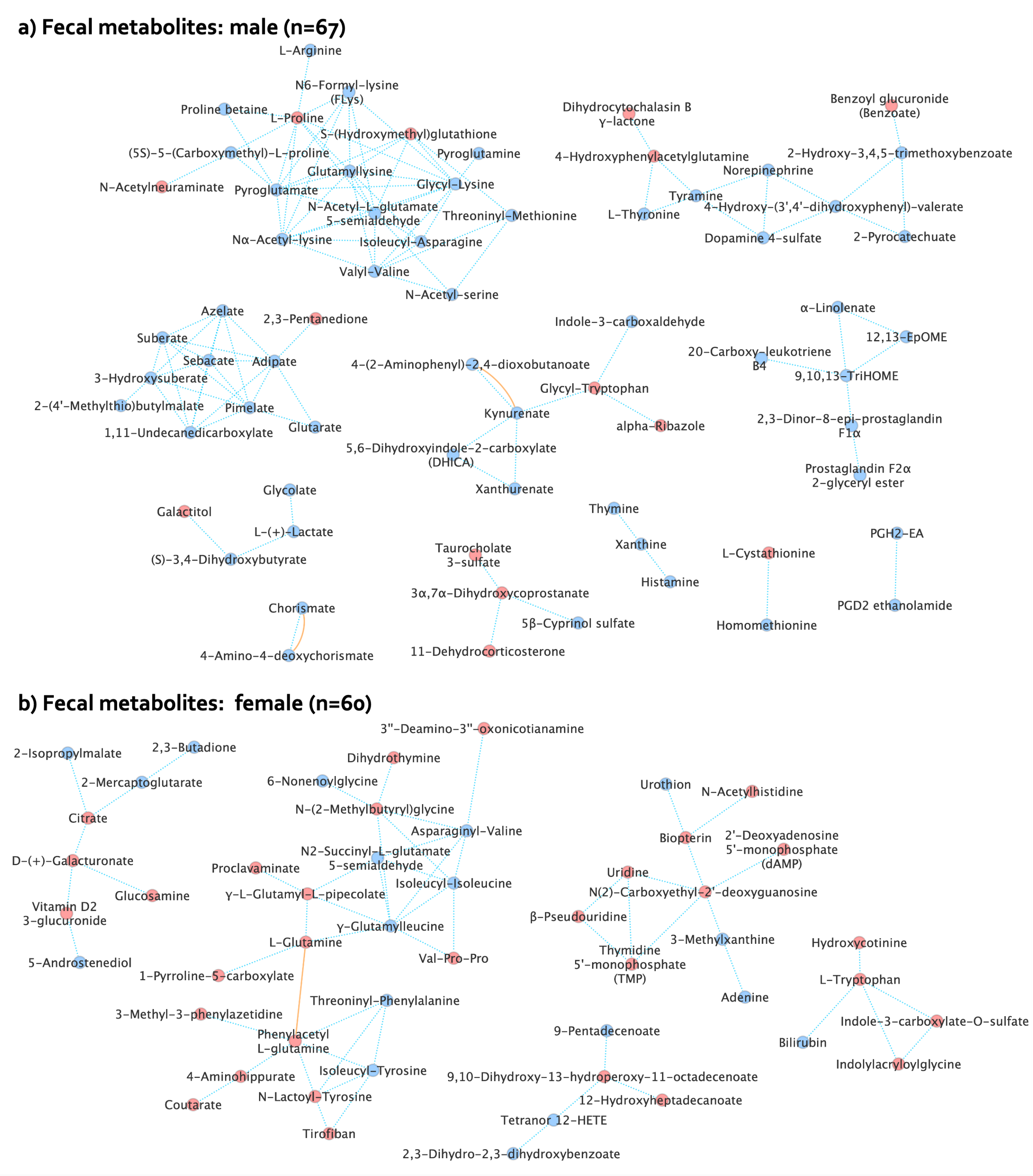

Supplementary Fig. 6. MetaMapp network view of sex-specific metabolites in feces comparing GF and CONV-R mice as determined by two-way ANOVA (adjusted p<0.05) and *post hoc* Tukey's HSD test (adjusted p<0.05).

(**a**) fecal metabolites altered in male but not female; (**b**) fecal metabolites altered in female but not male. Node colors: blue (CONV-R>GF); red (CONV-R<GF). Edges: blue dotted lines indicate chemical structural similarity (Tanimoto coefficient>0.7), while orange solid lines indicate biochemical relevance (KEGG reactant pair).

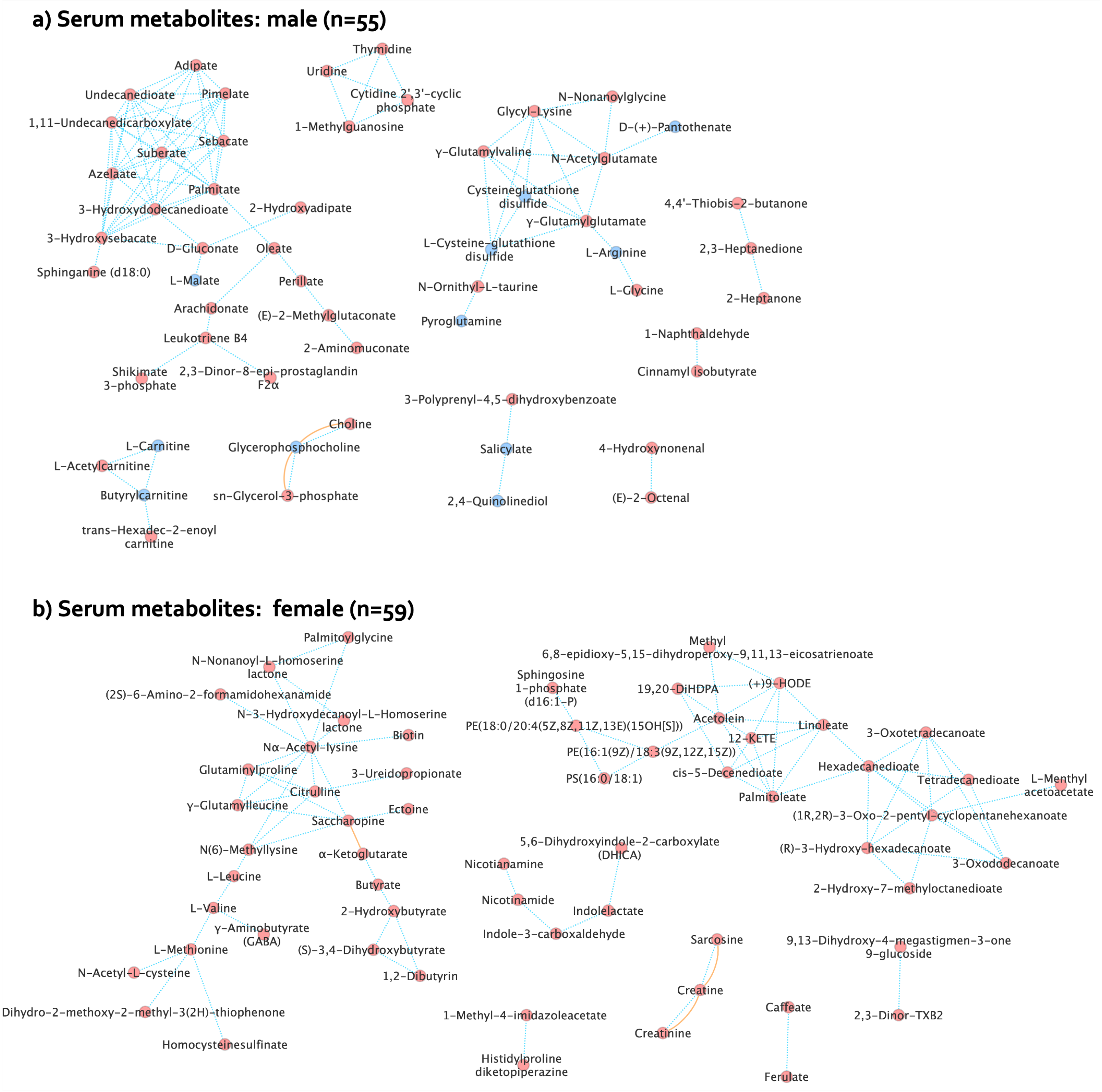

Supplementary Fig. 7. MetaMapp network view of sex-specific metabolites in blood sera comparing GF and CONV-R mice as determined by two-way ANOVA (adjusted p<0.05) and *post hoc* Tukey's HSD test (adjusted p<0.05).

(**a**) serum metabolites altered in male but not female; (**b**) serum metabolites altered in female but not male. For node colors, blue means CONV-R>GF, red means CONV-R<GF; for edges, blue dotted lines indicate chemical structural similarity (Tanimoto coefficient>0.7), and orange solid lines indicate biochemical relevance (KEGG reactant pair).

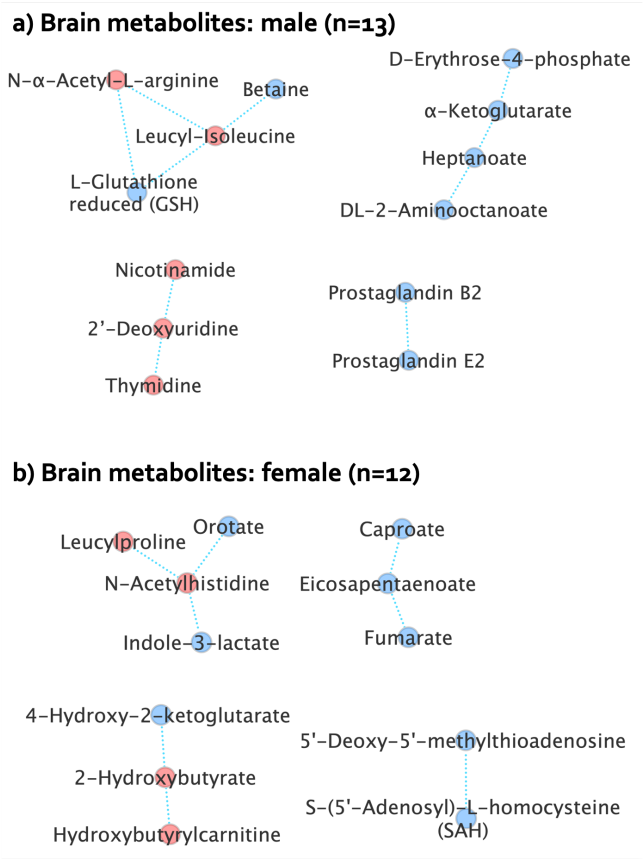

Supplementary Fig. 8. MetaMapp network view of sex-specific metabolites in cerebral cortical brain tissues comparing GF and CONV-R mice as determined by two-way ANOVA (adjusted p<0.05) and *post hoc* Tukey's HSD test (adjusted p<0.05).

(**a**) brain metabolites altered in male but not female; (**b**) brain metabolites altered in female but not male. For node colors, blue means CONV-R>GF, red means CONV-R<GF; for edges, blue dotted lines indicate chemical structural similarity (Tanimoto coefficient>0.7), and orange solid lines indicate biochemical relevance (KEGG reactant pair).

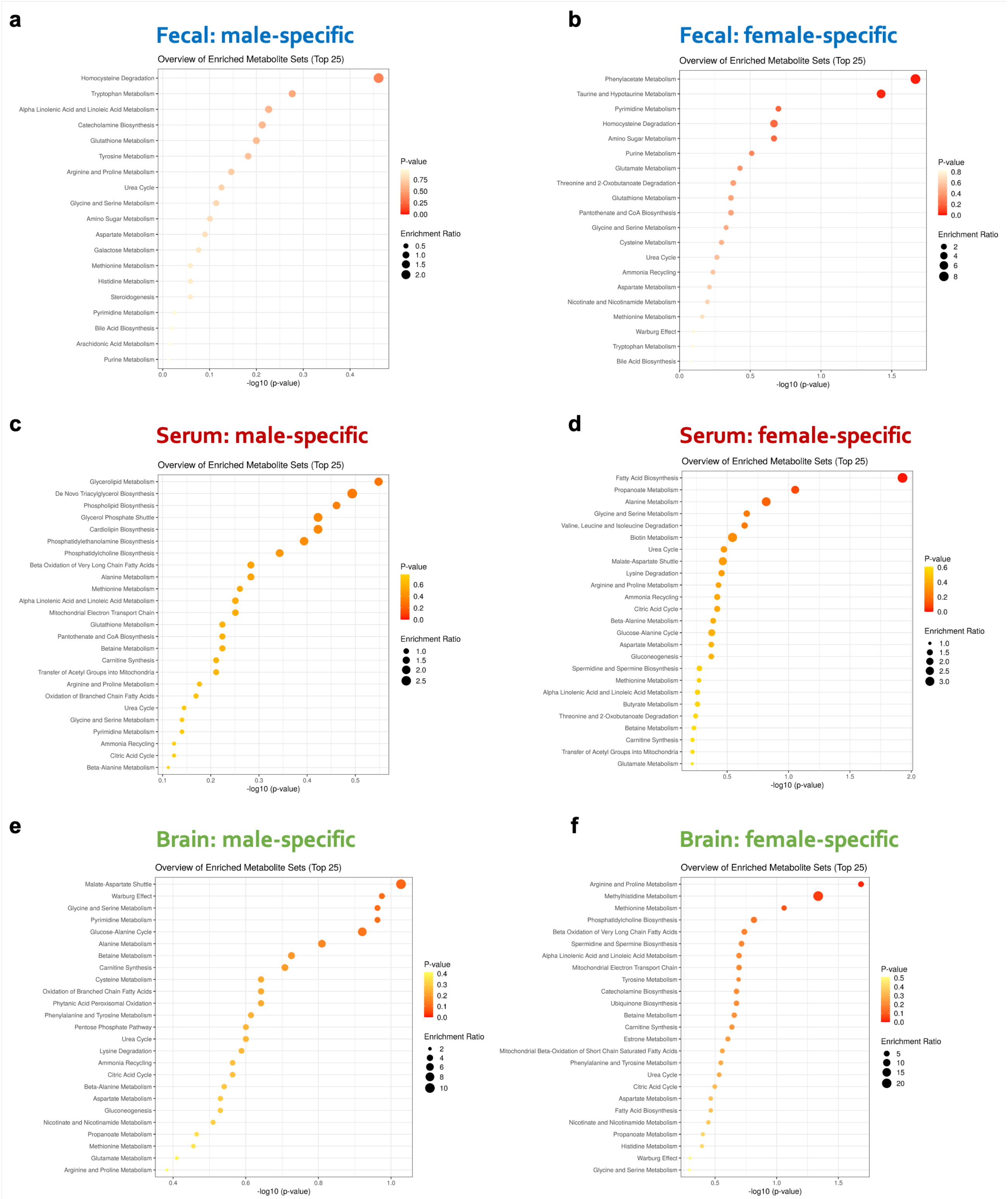

Supplementary Fig. 9. Metabolite set enrichment analysis (MSEA) of gender-specific metabolites for different sample matrices based on 99 *a priori* defined sets of metabolites (adjusted p-value<0.05). (**a**) male, fecal; (**b**) female, fecal; (**c**) male, sera; (**d**) female, sera; (**e**) male, cerebral cortical brain tissues; (**f**) female, cerebral cortical brain tissues.

Additional information

Settings of MS-FINDER 3.3 (running with Windows 10 OS)

for *in silico* compound annotation of ion features with an acquired tandem mass spectrum

Module	Parameter item / options	Value
**Method**	Spectral database search	yes
	… by in silico fragmentor	yes
	**Spectral database option**	
	Use internal experimental library	MassBank, GNPS, ReSpect
	Use in silico spectra of LipidBlast	Solvent type: HCOONH4
	User-defined DB	NULL
	**Precursor oriented option**	"Precursor oriented spectral search"
**Mass spectrum**	Mass tolerance type	"ppm"
	Mass tolerance (MS1)	10
	Mass tolerance (MS2)	10
	Relative abundance cutoff	1%
	Mass range	40-1000
**Formula finder**	LEWIS and SENIOR check	yes
	Isotopic ratio tolerance	5%
	Element ratio check	Common range (99.7%) covering
	Element selection	O, N, P, S
**Structure finder**	In silico MS/MS, tree depth	2
**Data source**	Local databases	HMDB (Human), Urine (Human), Saliva (Human), Feces (Human), Serum (Human), CSF (Human), SPMDB (Human), LipidMAPS (Lipids), ECMDB (E. coli), PubChem (Biomolecules)
	MINEs setting	Never use it.
	PubChem Online setting	Never use it.

Additional information

Random Forest Classification for Generating Variable Importance Plots (for Fig. 3a, 6a & 7a):

Hyperparameters, OOB estimate error rate, and confusion matrices

using R packages *randomForest* (ver. 4.16.4) and *rfPermute* (ver. 2.2)

**Feces** (533 metabolites)	library("randomForest", "rfPermute")<br>set.seed(2020)<br>rfPermute(as.factor(Group) ~ ., data = vip_fecCID, importance = TRUE, nrep = 1000)<br>Call:<br>randomForest(formula = as.factor(Group) ~ ., data = vip_fecCID, importance = TRUE)<br>Type of random forest: classification<br>Number of trees: 500<br>No. of variables tried at each split: 23<br>OOB estimate of error rate: 0%<br>Confusion matrix:<br>CONV-R GF class.error<br>CONV-R 12 0 0<br>GF 0 12 0
**Serum** (231 metabolites)	library("randomForest", "rfPermute")<br>set.seed(2020)<br>rfPermute(as.factor(Group) ~ ., data = vip_serCID, importance = TRUE, nrep = 1000)<br>Call:<br>randomForest(formula = as.factor(Group) ~ ., data = vip_serCID, importance = TRUE)<br>Type of random forest: classification<br>Number of trees: 500<br>No. of variables tried at each split: 15<br>OOB estimate of error rate: 0%<br>Confusion matrix:<br>CONV-R GF class.error<br>CONV-R 12 0 0<br>GF 0 12 0
**Brain** (58 metabolites)	library("randomForest", "rfPermute")<br>set.seed(2020)<br>rfPermute(as.factor(Group) ~ ., data = vip_brnCID, importance = TRUE, nrep = 1000)<br>Call:<br>randomForest(formula = as.factor(Group) ~ ., data = vip_brnCID, importance = TRUE)<br>Type of random forest: classification<br>Number of trees: 500<br>No. of variables tried at each split: 7<br>OOB estimate of error rate: 0%<br>Confusion matrix:<br>CONV-R GF class.error<br>CONV-R 12 0 0<br>GF 0 12 0

Random Forests typically do not overfit. But hyperparameter knobs may need fine tuning to best avoid overfitting dealing with noisy data. In this work, (1) **`nodesize.`** the minimum size for terminal nodes (leaves in the tree) was set as the default value of 1 (for classification); (2) **`maxnodes.`** the maximum number of nodes in each decision tree was set as default with no limit specified, the largest tree was to be fit subject to the constraints of **`nodesize`**. [4]

References

1 Tsugawa, H. *et al.* Hydrogen Rearrangement Rules: Computational MS/MS Fragmentation and Structure Elucidation Using MS-FINDER Software. *Anal Chem* **88**, 7946-7958, doi:10.1021/acs.analchem.6b00770 (2016).

2 Hughes, A. N. & Oxford, J. T. A lipid-rich gestational diet predisposes offspring to nonalcoholic fatty liver disease: a potential sequence of events. *Hepat Med* **6**, 15-23, doi:10.2147/hmer.S57500 (2014).

3 Belalcázar, A. D., Ball, J. G., Frost, L. M., Valentovic, M. A. & Wilkinson, J. t. Transsulfuration Is a Significant Source of Sulfur for Glutathione Production in Human Mammary Epithelial Cells. *ISRN Biochem* **2013**, 637897, doi:10.1155/2013/637897 (2014).

4 Bruce, P., Bruce, A. & Gedeck, P. *Practical Statistics for Data Scientists: 50+ Essential Concepts Using R and Python*. (O'Reilly Media, 2020).
