# Supplementary material for: High-coverage metabolomics uncovers microbiota-driven biochemical landscape of interorgan transport and gut-brain communication in mice
Source: Nat Commun. 2021 Oct 19;12:6000. doi: 10.1038/s41467-021-26209-8 (PMC8526691; doi:10.1038/s41467-021-26209-8)
Supplement: Supplementary file 2 — Description of Additional Supplementary Files [file 41467_2021_26209_MOESM2_ESM.docx]

**Description of Supplementary Files**

File Name: Supplementary Data 1

Description: List of microbial products, neurotransmitters, and canonical metabolites (n=423 compounds). This is for the in-house spectral library (both HESI positive and negative) established and used in this work. Successful detection of GF/CONV-R difference in feces, blood sera, and cortical brain tissues were also provided.

File Name: Supplementary Data 2

Description: Unique annotation list of 533 fecal metabolites altered in feces comparing GF and CONV-R mice. This file contains metabolite names, compound identifiers, mode of detection / annotation, fold change and statistics, and side notes for biological interpretation.

File Name: Supplementary Data 3

Description: Unique annotation list of 231 serum metabolites altered in feces comparing GF and CONV-R mice. This file contains metabolite names, compound identifiers, mode of detection / annotation, fold change and statistics, and side notes for biological interpretation.

File Name: Supplementary Data 4

Description: Unique annotation list of 58 cerebral cortical brain metabolites altered in feces comparing GF and CONV-R mice. This file contains metabolite names, compound identifiers, mode of detection / annotation, fold change and statistics, and side notes for biological interpretation.

File Name: Supplementary Data 5

Description: ChemRICH results from all altered fecal metabolites for probing fecal metabolome changes on the compound class level.

File Name: Supplementary Data 6

Description: ChemRICH results of fecal metabolome changes: assignment for individual metabolites.

File Name: Supplementary Data 7

Description: Quantitative pathway enrichment analysis (qMSEA) results of all altered fecal metabolites for probing fecal pathway changes.

File Name: Supplementary Data 8

Description: ChemRICH results from all altered serum metabolites for probing serum metabolome changes on the compound class level.

File Name: Supplementary Data 9

Description: ChemRICH results of serum metabolome changes: assignment for individual metabolites.

File Name: Supplementary Data 10

Description: Quantitative pathway enrichment analysis (qMSEA) results of all altered serum metabolites for probing serum pathway changes.

File Name: Supplementary Data 11

Description: Gender-specific metabolites of GF/CONV-R difference (only altered in either male or female groups) in feces, blood sera, and cerebral cortical brain tissues based on two-way ANOVA and Tukey’s HSD test.
